# Structural analysis of temperature-dependent alternative splicing of HsfA2 pre-mRNA from tomato plants

**DOI:** 10.1080/15476286.2021.2024034

**Published:** 2022-02-07

**Authors:** Patrizia Broft, Remus Rosenkranz, Enrico Schleiff, Martin Hengesbach, Harald Schwalbe

**Affiliations:** a Institute for Organic Chemistry and Chemical Biology, Goethe University, Frankfurt am Main, Germany; b Department of Biosciences, Molecular Cell Biology of Plants, Goethe University, Frankfurt am Main, Germany

**Keywords:** Alternative splicing in plants, NMR spectroscopy, RNA, in-line probing

## Abstract

Temperature-dependent alternative splicing was recently demonstrated for intron 2 of the gene coding for heat shock factor HsfA2 of the tomato plant *Solanum lycopersicum*, but the molecular mechanism regulating the abundance of such temperature-dependent splice variants is still unknown. We report here on regulatory pre-mRNA structures that could function as regulators by controlling the use of splice sites in a temperature-dependent manner. We investigate pre-mRNA structures at the splice sites of intron 2 of the gene coding for HsfA2 from *S. lycopersicum* using NMR- and CD-spectroscopy as well as in-line probing. The pre-mRNA undergoes conformational changes between two different secondary structures at the 3ʹ splice site of the intron in a temperature-dependent manner. Previously, it was shown that three single nucleotide polymorphisms (SNPs) in intron 2 of the HsfA2 pre-mRNA affect the splicing efficiency of its pre-mRNA and are linked to the thermotolerance in different tomato species. By comparing pre-mRNA fragments of the tomato species *S. lycopersicum* and *S. peruvianum*, we show that these SNPs result in substantial structural differences between the pre-mRNAs of the two species.

## Introduction

Plants are sessile and thus have to adapt their metabolism in response to external biotic and abiotic stresses, including salinity, drought, wind, or temperature. Due to climate change, plants are increasingly exposed to abiotic stress signals such as long-term increased temperature to which they have to react [1]. Plants can respond to temperature stress by a multitude of mechanisms including alternative splicing (AS) [2–6]. The heat shock transcription factor HsfA2, a key regulator in response to heat stress in tomato [7], is significantly upregulated under heat stress [8] and alternatively spliced in a temperature range of 30–45°C in the tomato plant *Solanum lycopersicum* [9].

Heat shock factors are essential for plants to survive, as they act as transcriptional repressors or activators to regulate the expression of heat shock proteins, which in turn act as chaperones to suppress misfolding of proteins in temperature-stressed cells [10]. It is therefore important to understand how pre-mRNA splicing of heat shock factors is regulated.

**Figure 1.**
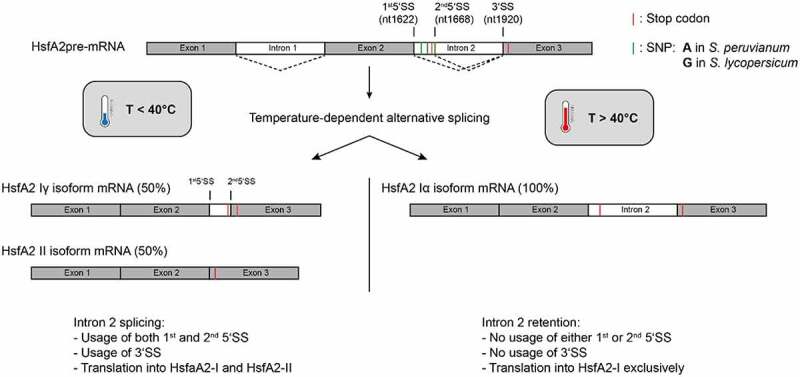
Schematic representation of alternative splicing of the HsfA2 pre-mRNA from *S. lycopersicum* at different temperatures. Intron 2 of the pre-mRNA is alternatively spliced depending on the temperature. Exons are shown in grey and introns are shown in white. Different splicing reactions are symbolized by dashed lines. The position of the 5‘- and 3‘-splice site(s) (SS) of intron 2 are marked with black lines. The splice variants HsfA2-Iγ and HsfA2-II are mainly generated below 40°C by using alternative 5 ‘splice sites of intron 2. Splice variant HsfA2-Iγ results from the usage of the second 5’SS and splice variant HsfA2-II results from the usage of the first 5’SS. Above 40°C, there is predominantly no more splicing of intron 2 (intron retention). No splice sites are used. The position of the SNPs (three G’s in *S.lycopersicum* vs. three A’s in *S. peruvianum*) are marked in green.

The gene encoding HsfA2 is transcribed from chromosome 8 to yield a 2873nt pre-mRNA. There are 2 introns located in this pre-mRNA (Figure 1), both of which have been shown to be fully or partially retained in alternatively spliced isoforms [9]. Both of these introns contain stop codons. Utilization of these stop codons leads in case for intron 2 to the translation of a shorter protein isoform upon partial or full intron retention. Retention or partial retention of intron 1 occurs rarely, resulting in HsfA2-III mRNA-isoforms [9] (not shown in Figure 1). These isoforms are subject to degradation and for this reason it is believed that the HsfA2-III protein does not exist in tomato plants [9]. Intron 2 is primarily subject to alternative splicing. Three main splicing isoforms of the HsfA2 pre-mRNA are formed, by full splicing of intron 1 and full, partial or no splicing of intron 2. Isoform HsfA2-II uses the canonical splice sites and thus results in the shortest splice variant. The complete or partial retention of intron 2 results in the two splicing variants, HsfA2-Iα and -Iγ. The relative abundance of these three isoforms is temperature-dependent [9]. The splice variant HsfA2-Iα is most abundant for severe heat stress at temperatures above 40°C. The shorter splice variants, HsfA2-Iγ and HsfA2-II, result from splicing using different 5ʹ splice sites of intron 2 and are mainly formed at temperatures below 40°C. The distribution of alternatively spliced mRNAs shifts with the temperature from mainly HsfA2-II at 30°C to both -II and -Iγ up to 40°C. At temperatures higher than 40°C, -Iα becomes the major product.

In general, regulation of alternative splicing is achieved by the exclusive selection of the respective splice sites. On a molecular level, this selection can occur via a number of different mechanisms, two of which are (1) RNA elements such as splicing enhancers or splicing silencers, and (2) proteins (such as polypyrimidine tract-binding proteins (PTBs) or other splicing regulators) that interact with splice sites or the abovementioned RNA elements [11]. These interactions then allow the recruitment of spliceosomal ribonucleoprotein particles (U1 snRNP to the 5’SS, and U2 snRNP to the 3’SS) to the selected splice sites, which are used to generate the mRNA isoforms.

The protein isoforms generated from HsfA2-I and -II have been shown to exhibit differences in their subcellular localization. Furthermore, it was reported that intron 2 of *S. peruvianum*, although it does not have the splice variant HsfA2-Iγ, is spliced more efficiently than intron 2 of *S. lycopersicum*. The reason for this different splicing behaviour was previously attributed to three non-consecutive intronic single nucleotide polymorphisms (SNPs) located near the 5ʹ splice site of intron 2 (Figure 1, highlighted in green). The SNPs in the HsfA2 pre-mRNA are three G's in *S. lycopersicum* as opposed to three A’s in *S. peruvianum* (A1645G, A1651G and A1669G) (Supplementary Figure S1) [9].

Here, we set out to investigate whether pre-mRNAs can adopt different stable structures at different temperatures as a molecular determinant for temperature-dependent splice variations. Such structural switch in plants would be reminiscent to RNA thermometers identified in prokaryotes. There, RNA thermometers can regulate gene expression by local change of secondary structure as a function of temperature. They typically control the translation by blocking ribosomal binding sites through their structure and exposing them when the temperature rises [12]. In analogy to RNA thermometers in bacteria, we hypothesized that the change in splicing due to temperature changes for Intron 2 of the HsfA2 gene from *S. lycopersicum* could depend on the availability or sequestration of the corresponding splice sites. We further hypothesized that the underlying mechanism may require the presence of certain RNA structures to direct the decision for splicing between the splice sites within this gene. The assumption that specific RNA structures regulate gene expression in plants is not unprecedented, since an RNA thermoswitch was recently identified in *Arabidopsis* that regulates the translation of PIF7 mRNA [13], and other processes similar to those in prokaryotes have been shown to also regulate alternative splicing [14]. Furthermore, an RNA thermosensor was found in yeast which regulates the alternative use of 3’SS triggered by heat shock [15]. RNA thermometers that regulate temperature-dependent splicing in plants, however, have not been reported. In general, the molecular mechanisms of temperature-dependent alternative splicing of genes are poorly understood [16].

In order to decipher possible splicing regulation by temperature-dependent RNA conformational switching, we analysed and compared constructs encompassing the 5ʹ and 3ʹ splice sites of the HsfA2 intron 2 from the tomato species *S. peruvianum* with HsfA2 intron 2 from *S. lycopersicum*. We investigated pre-mRNA sequences at the 3ʹ and 5ʹ splice site of intron 2 at various temperatures using inline-probing, circular dichroism (CD) and nuclear magnetic resonance (NMR) spectroscopy. Our studies show that the RNA structures at the 5ʹ splice site of *S. lycopersicum* and *S. peruvianum* do not undergo significant temperature-dependent conformational changes, but differ in terms of their thermal stability and structure as a consequence of three SNPs. In stark contrast, however, the pre-mRNA at the 3ʹ splice site from *S. lycopersicum* adopts different long-lived conformations in a temperature-dependent fashion, suggesting the importance of RNA sequence and conformation in the splice site selection of pre-mRNA introns.

## Results

### Inline-probing and CD analysis of pre-mRNA structures at the 5ʹ splice site

**Figure 2.**
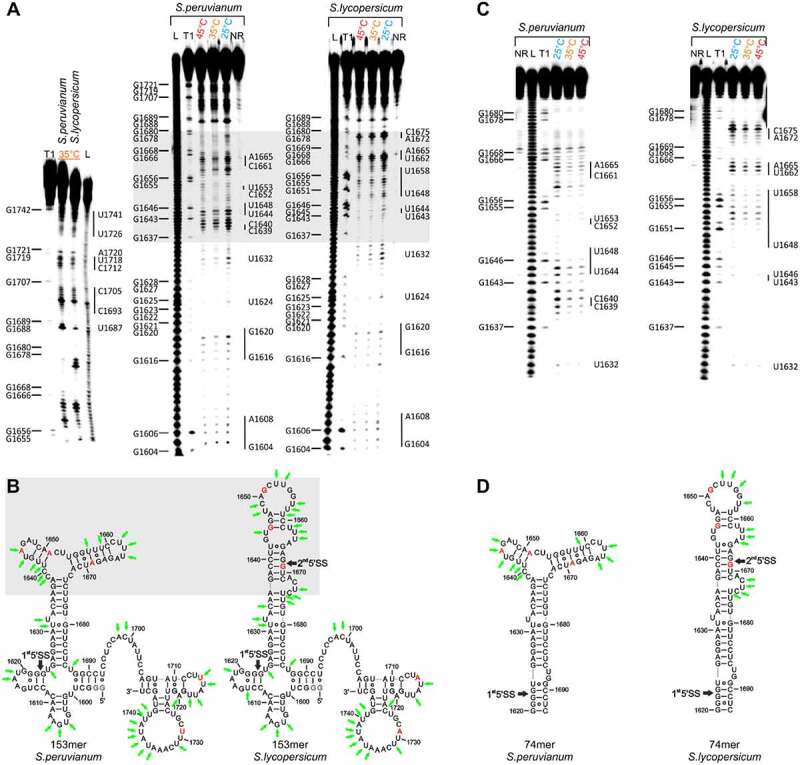
Temperature-dependent in-line probing analysis and structural models of the RNA constructs at the 5’SS of intron 2 of the HsfA2 pre-mRNA. (a) Left: In-line probing analysis of 153mer*^S.peruv^* and 153mer*^S.lyco^* at 35°C. Right: Temperature dependent in-line probing analysis in a range of 25–45°C. 153mer*^S.peruv^* and 153mer*^S.lyco^* show distinctly different cleavage patterns for the nucleotide range 1636–1676 (highlighted in grey). NR: unreacted control, T1: RNaseT1 digest resulting in G-specific cleavage, L: alkaline digest resulting in unspecific cleavage. G residues are assigned on the left side of each gel. Nucleotides or nucleotide sequences that are not base-paired in the structural model are assigned to the right-hand side of each gel. (b) Structural model derived from analysis of in-line probing of 153mer*^S.peruv^* and 153mer*^S.lyco^*, and including Mfold prediction. SNPs are highlighted in red. The 1^st^ and (2^nd^) 5ʹ splice sites (5’SS) are marked with black arrows. The structural difference between the two molecules is highlighted in grey. Nucleotide positions at which a relatively large amount of spontaneous cleavage was found in the in-line probing experiment are marked with green arrows. (c) In-line probing analysis of 74mer*^S.peruv^* and 74mer*^S.lyco^* in a temperature range of 25–45°C. Lanes designated NR, T1, and L identify RNA samples loaded after subjecting to no reaction, partial digestion with RNase T1, or partial digestion with alkali, respectively. Bands corresponding to RNase T1 cleavage after G residues are assigned on the right side of each gel. Nucleotides or nucleotide sequences that are not base-paired in the structural model are assigned to the left-hand side of each gel. (d) Structural model for 74mer*^S.peruv^* and 74mer*^S.lyco^* derived from analysis of in-line probing, and including Mfold prediction. The 1^st^ and (2^nd^) 5ʹ splice sites (5’SS) are marked with black arrows. The SNPs are highlighted in red. Nucleotide positions at which a relatively large amount of spontaneous cleavage was found in the in-line probing experiment are marked with green arrows.

We first designed, synthesized and investigated an RNA covering the 5ʹ splice site of HsfA2 intron 2. Here, we characterized the temperature-dependence of the conformation of 153nt long RNA constructs from the species *S. lycopersicum* and *S. peruvianum* (153mer*^S.lyco^ and* 153mer*^S.peru^*^v^). Both 153mers cover the sequence range between nt1598-nt1747 of the pre-mRNA, contain the 1^st^ (and 2^nd^) 5’SS between nt1622/1623 (and nt1668/1669) of intron 2 and differ in their sequence in 5 non-consecutive SNPs at positions 1645, 1651, 1669, 1714 and 1729 (3A’s/3G’s and 2U’s/2A’s). For the SNPs A1645G, A1651G, and A1669G it was previously reported that these are decisive for the different splicing efficiency of intron 2 of the two plant species [9]. We first examined the RNA sequences using in-line probing in the alternative splicing relevant temperature range of 25°C-45°C (Figure 2(a)) and derived secondary structure models (Figure 2(b)).

The analysis showed that 153mer*^S.peruv^* and 153mer*^S.lyco^* were structurally very similar except for the range between nucleotides 1636–1676. Whereas the SNPs U1714A and U1729A are located in structurally similar areas of both RNAs (Figure 2(b)), we found that the remaining three splicing-relevant SNPs A1645G, A1651G and A1669G lead to significant structural differences in the range of nt1636-nt1676 between the RNAs from *S. lycopersicum* and *S. peruvianum*. Our secondary structure model showed that in each case, the splice sites (nt 1622/1623 for 153mer*^S.lyco^* and 153mer*^S.peruv^*, and nt 1668/1669 for 153mer*^S.lyco^*) were base-paired, which could sequester these sequences from being utilized for splicing. In either case, the sequences did not significantly change their conformation with the temperature at which the in-line probing analysis was performed.

For the pre-mRNA sequences investigated above, the structural models derived from inline-probing were compared with the Mfold structure predictions of larger pre-mRNA fragments (617nt; nt1439-nt2055) (Supplementary Figure S2), revealing very good agreement for the region from nt1625-nt1686 for both 153mers. In order to characterize the structural differences in more detail, we designed 74mers which contain the sequence region of nt1620-1693 of the pre-mRNAs from *S. lycopersium* and *S. peruvanium* (74mer*^S.lyco^* and 74mer*^S.peruv^*) and which contain the 5ʹ splice site(s) as well as the three non-consecutive SNPs that are decisive for the structural difference between the RNAs of the two species.

We examined the 74mer*^S.lyco^* and 74mer*^S.peruv^* using in-line probing in a temperature range of 25°C–45°C (Figure 2(c)). The cleavage pattern of both 74mer RNAs agreed very well with the corresponding region of the probing pattern of the 153mer RNAs (Figure 2(a, c)). Upon variation of the probing temperature, we find that both RNAs do not undergo significant conformational changes in this temperature regime.

In contrast to the 153mer*^S.peruv^*, we did, however, observe spontaneous cleavage for nucleotides 1668 and 1669 for 74mer*^S.peruv^*. Since these bands are also visible in the negative control sample, we deem these cleavages not relevant for our structural analysis. We again created structural models based on the probing data as well as the Mfold prediction (Figure 2(d)). While the structural model for 74mer*^S.lyco^* agreed very well with the experimental data, such agreement was only partially observed for the structural model of 74mer*^S.peruv^*. The thus derived structure model for 74mer*^S.peruv^* does not explain all experimental data. Some sequence regions including nucleotides nt1666-nt1667, nt1657-nt1660 and nt1641-1642 showed increased spontaneous cleavage, which cannot entirely be explained based on the structural model. These nucleotides would be located in helices that contain the SNPs. On the one hand, the helix, which contains nt 1666–1667 and nt 1657–1660, may be of limited stability due to two G-U basepairs surrounded by A-U basepairs. Similarly, the helix which includes nt1641-1642, would contain only three basepairs. Therefore, formation of these these motifs may be weak and/or transient. In addition, the deviation could be explained by structural heterogeneity. There could be an alternative structure or structures (for a possible alternative structure see Supplementary Figure S3). It should also be noted that the same possible structural heterogeneity is visible not only in 74mer*^S.peruv^* but also in the less relevant 153mer*^S.peruv^*. This potential structural heterogenity however only occurs in a region which does not contain the SS of 74mer*^S.peruv^*.

**Figure 3.**
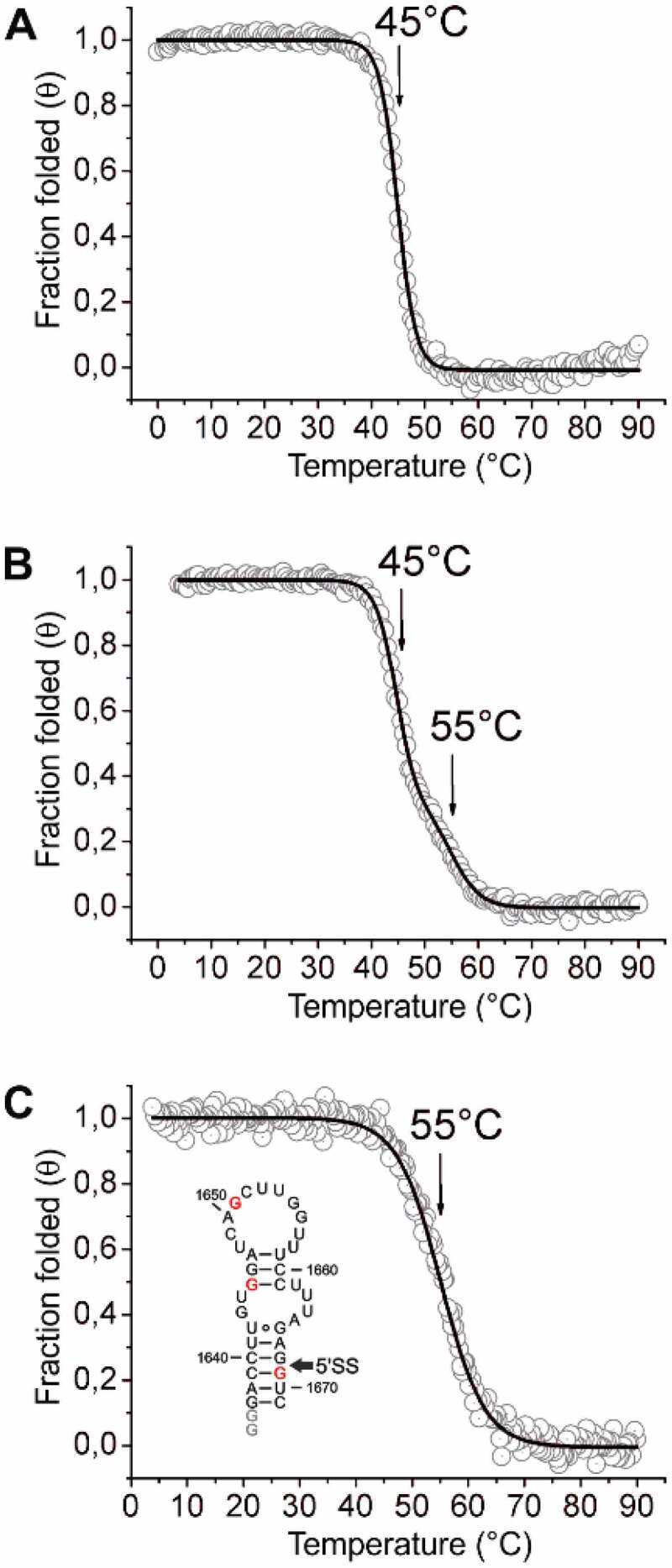
CD-melting analysis of relevant RNA constructs at the 5’SS of intron 2 of the HsfA2 pre-mRNA. CD-melting curves of 74mer*^S.peruv^*, 74mer*^S.lyco^* and 37mer*^S.lyco^* in 25 mM potassium phosphate buffer (pH 6.3) at a concentration of 10 µM. Melting points are marked with arrows. (a) CD melting curve of 74mer*^S.peruv^*. (b) CD melting curve of 74mer*^S.lyco^*. (c) CD melting curve of 37mer*^S.lyco^*. The structure of the 37mer is shown in the inset with SNPs highlighted in red and non-native G nucleotides highlighted in grey. The second splice site (5’SS) is marked with an arrow. Because of the ambiguity of the determination of ΔH and ΔS from the CD melting data, we refrain from extracting these parameters here.

In order to assess the structural stability of these RNAs, CD melting curves were recorded and melting points determined (Figure 3). For 74mer*^S.peruv^* a melting point of 45°C was observed (Figure 3(a)), whereas 74mer*^S.lyco^* showed two independent melting points of 45°C and 55°C (Figure 3(b)). To clarify the second transition in the melting curve of 74mer*^S.lyco^*, we also tested a smaller 37mer RNA segment of *S. lycopersicum* (37mer*^S.lyco^*, nt1637-nt1671), which contains the SNPs of three Gs as well as the splice site at position 1668/1669 (Figure 3(c)). The melting points of the shorter construct coincides with the higher melting point of the longer construct at 55°C, suggesting that this melting transition is linked to unfolding of this smaller sequence segment. From the CD data, we derive that the 74mer*^S.lyco^* containing the three G-SNPs has an overall more stable structure than the 74mer*^S.peruv^* containing the three A-SNPs, and that the second splice site of 74mer*^S.lyco^* is located in a thermally more stable structural region (melting point 55°C) compared to the first splice site (melting point 45°C).

### NMR analysis of pre-mRNA structures at the 5ʹ splice site

**Figure 4.**
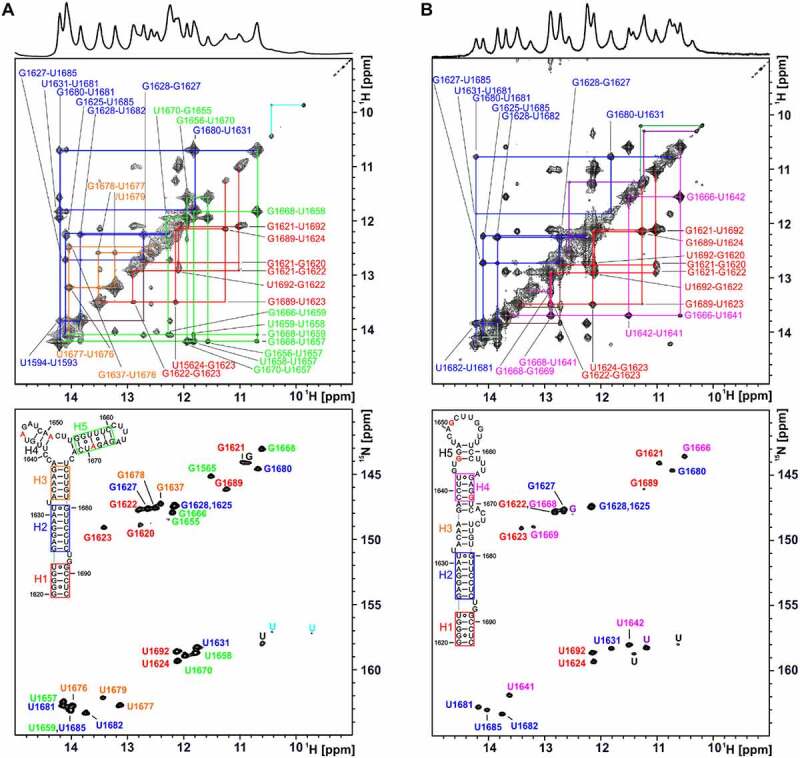
NMR analysis of relevant RNA constructs at the 5’SS of intron 2 of the HsfA2 pre-mRNA. A+B) ^1^H,^1^H-NOESY and ^1^H,^15^N-TROSY spectrum of 74mer*^S.peruv^* (A) and 74mer*^S.lyco^* (B) annotated with assignment. Imino-proton correlations between consecutive base pairs (imino walks) are shown in different colours in the NOESY spectra. The secondary structures determined experimentally are shown in the inset. NMR signals and corresponding structural elements are colour coded. RNA NMR spectra were recorded in 25 mM potassium phosphate buffer (pH 6.3) with 8% D_2_O at 10°C. (a) Top: NOESY spectrum of 74mer*^S.peruv^* (1.2 mM) recorded at 700 MHz with 8192 × 744 points and 128 scans. Bottom: TROSY spectrum of ^15^N GU-labelled 74mer*^S.peruv^* (140 µM) at 700 MHz with 1024 × 160 points and 16 scans. (b) Top: NOESY spectrum of 74mer*^S.lyco^* (930 µM) recorded at 800 MHz with 7996 × 800 points and 96 scans. Bottom: TROSY spectrum of ^15^N GU-labelled 74mer*^S.lyco^* (50 µM) recorded at 700 MHz with 1024 × 160 points and 16 scans.

^1^H,^1^H NOESY and ^1^H,^15^N TROSY spectra of unlabelled and ^15^N-labelled samples were recorded to sequentially assign the imino ^1^H,^15^N resonances of 74mer*^S.lyco^* and 74mer*^S.peruv^* (Figure 4(a, b)) at a temperature of 10°C. This low temperature is required to detect weak base pairs, in particular the four to five GU base pairs in both RNAs predicted from the structural model.

For both 74mers, most of the imino signals and helical segments of the secondary structures could be assigned (cf. coloured boxes in the secondary structure insets in Figure 4). The assignment of the spectra clearly shows that the structures of 74mer*^S.lyco^* and 74mer*^S.peruv^* are only partially identical. The identically structured segments include the helix H1 at the end of the RNAs (coloured red, Figure 4) as well as the directly adjacent helix H2 (coloured blue, Figure 4). Structurally different regions could also be assigned (helix H5 and helix H4, coloured green and pink, Figure 4). With a size of 74 nucleotides, the two investigated RNAs are large for NMR spectral analysis. As a consequence, signal overlap cannot be avoided. In order to support the assignment in cases of such overlap, we thus devised shorter fragments of the 74mer RNAs. For example, in the NOESY spectrum of 74mer*^S.peruv^*, (Figure 4(a)) the cross peaks of the GU base pairs G1680-U1631 from helix H2 and G1668-U1658 from helix H5 as well as other cross peaks and one signal in the TROSY spectrum of these helices overlap (coloured green and blue). This is not the case for the spectra of the 74mer*^S.lyco^*, as can be seen for an RNA fragment comprising nt1655-nt1671, whose signal assignment is shown in green (Figure 4(a) and Supplementary Figure S4). A second example is the signals of G1622 and G1668 that overlap in the TROSY spectrum of 74mer*^S.lyco^*. Again, this overlap was resolved by investigating a fragment composing nt1637-nt1672 (Supplementary Figure S5). Only one signal overlay, the overlay of G1628 and G1625, which is present in the assignment of the TROSY spectra of 74mer*^S.lyco^* and 74mer*^S.peruv^*, could not be clearly resolved. In general, however, RNA assignment requires that iminos are involved in stable base pairs at a temperature of 30°C (Supplementary Figure S6).

In order to also assess the thermal stability even of individual base pairs in 74mer*^S.peruv^* and 74mer*^S.lyco^* by NMR, ^1^H-1D imino proton spectra were recorded at temperatures in the range of 10–45°C (Supplementary Figure S7). These spectra show that helix H2 is stable in both RNAs and that helix H3 in 74mer*^S.peruv^* (Supplementary Figure 7A) and helix H4 in 74mer*^S.lyco^* (Supplementary Figure 7B) are also relatively stable over the temperature range of these experiments. Furthermore, it is clear from the spectra that the first splice site of both RNAs is located in an equally unstable structural region (helix H1) and that the second splice site of 74mer*^S.lyco^* is located in a relatively stable structural region (helix H4), which is in line with the CD data.

Under NMR conditions, we thus find evidence of a temperature-induced destabilization of the 1^st^ 5’SS of the RNAs (melting), but no evidence of a switch-like conformational change. The similar decreasing stability of the 1^st^ 5’SS as the temperature rises cannot explain the more frequent intron retention with increasing temperature, nor can it explain the diverging splicing efficiency of 74mer*^S.peruv^* and 74mer*^S.lyco^*.

### Inline-probing and CD analysis of pre-mRNA structures at the 3ʹ splice site

**Figure 5.**
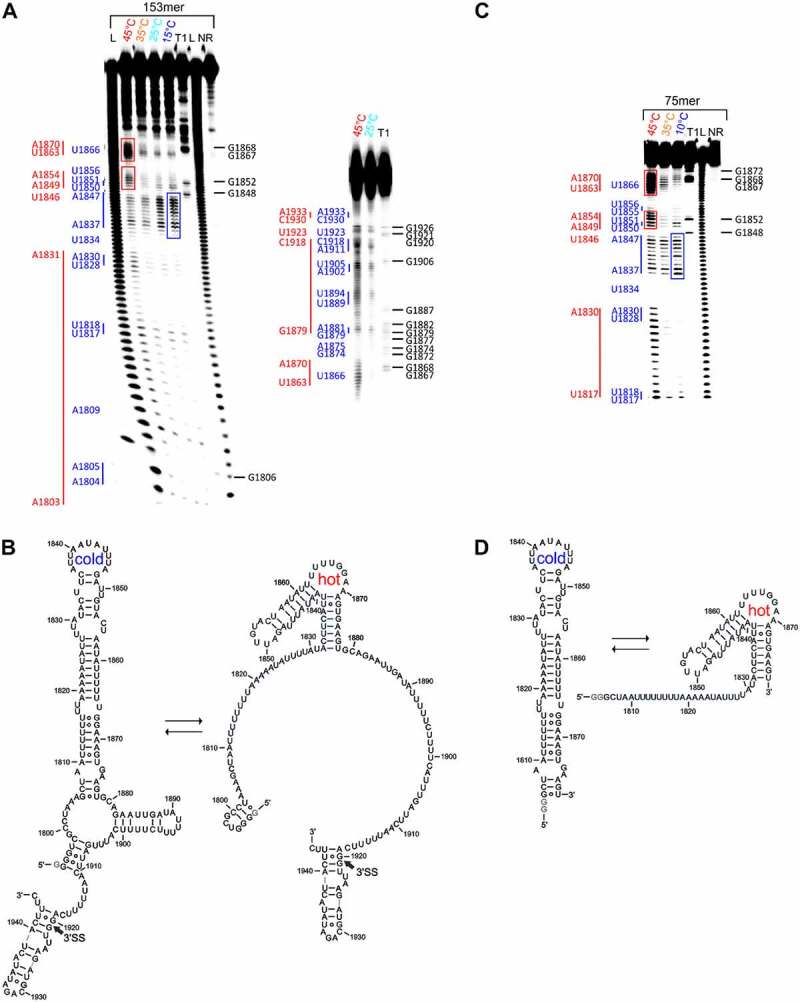
Temperature-dependent in-line probing analysis and structural models of the RNA constructs at the 3’SS of intron 2 of the HsfA2 pre-mRNA. (a) Left: In-line probing analysis of 153mer in a temperature range of 15–45°C. Lanes designated NR, T1, and L identify RNA samples loaded after subjecting to no reaction, partial digestion with RNase T1, or partial digestion with alkali, respectively. Bands corresponding to RNase T1 cleavage after G residues are assigned on the right side of each gel. Right: Re-run of the same samples as in the gel on the left to better resolve the region above ~ nt 1860 (b) Structure models of 153mer *S.lycopersicum*. The splice site (3’SS) is marked with an arrow, non-native G nucleotides are highlighted in grey. (c) In-line probing analysis of the 75mer in a temperature range of 10–45°C. Lanes designated NR, T1, and L identify RNA samples loaded after subjecting to no reaction, partial digestion with RNase T1, or partial digestion with alkali, respectively. Bands corresponding to RNase T1 cleavage after G residues are assigned on the right side of the gel. Nucleotides or nucleotide sequences that are not base-paired in the structural model are assigned to the left side of the gel in blue for the cold conformation and in red for the hot conformation. Intense bands of the hot conformation are marked with red boxes. Prominent bands of the cold conformation are marked with a blue box. (d) Structure model of the cold conformation (left) and hot conformation (right) of 75mer. Non-native G nucleotides are highlighted in grey.

In a second step, we investigated the 3ʹ splice site of the RNA derived from *S. lycopersicum*. To identify whether there are temperature-dependent conformational changes, we conducted in-line probing of a 153 nt long pre-mRNA fragment (nt1794-1946) in a temperature range from 15°C to 45°C (Figure 5(a)).

The inline probing analysis shows that the sequence range nt1838-1847, which is subject to strong spontaneous cleavage at a low temperature of 15°C (Figure 5(a), blue box), is cleaved less efficiently at the higher temperature of 45°C, although other sequence areas nt1850-1854 and nt1862-1871 (Figure 5(a), red boxes) are subject to much stronger spontaneous cleavage at increased temperature.

Since we carefully ensured statistical cleavage of the RNA (as evidenced by the prominent band of full-length RNA), this points to two distinct conformations adopted by this RNA at different temperatures. The temperature-dependent change in conformation is clearly visible on the probing gel, especially in the nt range 137–147 (Figure 5(a)). The band intensities in this area decrease sharply as the temperature rises relative to other band intensities from other areas (e.g. nt1863-1870) (Figure 5(a), blue and red boxes). With the different base-pairing patterns indicated by the probing data, we were able to generate a structural model for the conformational switch between the two temperatures. (Figure 5(b)).

Since this RNA contains the 3ʹ splice site located between nt1920 and nt1921, we furthermore can assess whether this sequence is accessible in the two conformations. The 3ʹ splice site is predominantly base-paired in the temperature range of 25–45°C, but tends to be more exposed when the temperature increases (Figure 5(a)). Strikingly, however, an element between nucleotides 1806 and 1878 of the RNA adopts different conformations at different temperatures. We were able to detect a similar behaviour for a 143nt long RNA which comprises the sequence region nt1806-1946 of the pre-mRNA (Supplementary Figure S8).

To further define the parts of the pre-mRNA that are involved in the temperature-dependent conformational change, we devised a 75mer construct based on the results of the probing experiments for the 153mer (Figure 5(c)). This 75mer encompasses the sequence range of nt1806-1878 of the pre-mRNA and, according to the structure prediction, covers the area where temperature-dependent conformational changes of the 153mer and 143mer pre-mRNA occur. In-line probing analysis of the 75mer showed nearly identical spontaneous cleavage patterns and the same conformational change as the 153mer for the selected sequence range (Figure 5(a, c)). This finding strongly suggests that the conformational change is limited to this sequence element of the pre-mRNA, and is not affected by other elements present within the 153mer. In order to better describe the different conformations, we derived structural models of the 75mer for which we coin the terms ‘cold’ and ‘hot’ conformation, respectively (Figure 5(d)).

**Figure 6.**
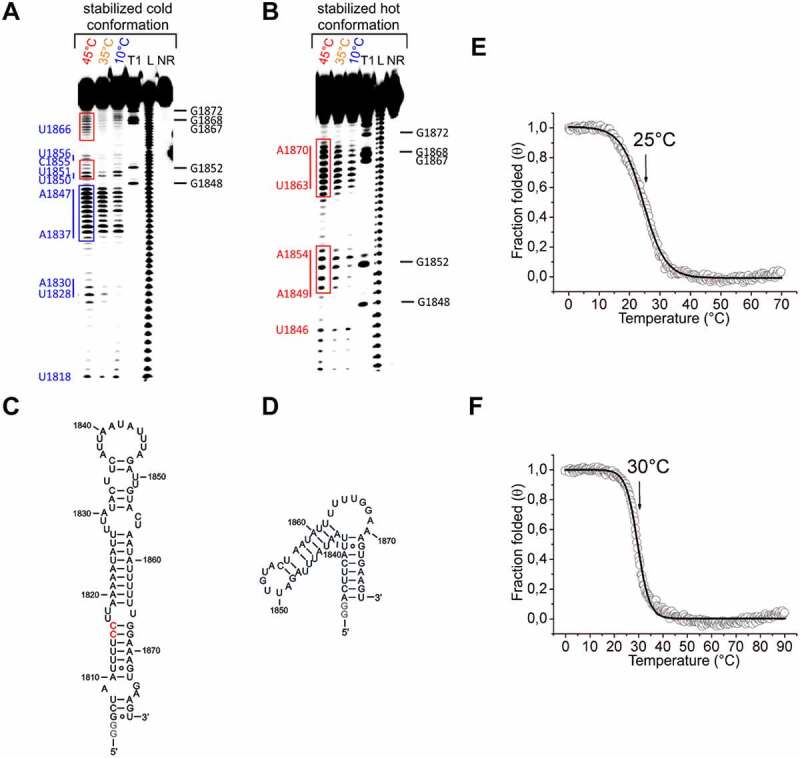
Temperature-dependent in-line probing analysis of the stabilized cold and hot conformation and CD analysis of the 75mer and its stabilized cold conformation. A+B) In-line probing analysis of the stabilized cold (a) and stabilized hot conformation (b) of the 75mer in a temperature range of 10–45°C. Lanes designated NR, T1, and L identify RNA samples loaded after subjecting to no reaction, partial digestion with RNase T1, or partial digestion with alkali, respectively. Bands corresponding to RNase T1 cleavage after G residues are assigned on the right side of each gel. Nucleotides or nucleotide sequences that are not base-paired in the structural models (C+D) are assigned to the left side of each gel (in blue for the stabilized cold conformation and in red for the stabilized hot conformation). The position of prominent bands of the stabilized hot conformation are marked with red boxes (B). The same position of these bands is marked with red boxes in the probing gel of the stabilized cold conformation (A). The blue box highlights signals of the stabilized cold conformation (A), which appear more intense at 45°C compared to the non-stabilized variant (Figure 5(c)). C+D) Structure model of the stabilized cold (c) and stabilized hot conformation (d) of the 75mer. The stabilizing mutations (2xC) of the cold conformation of the 75mer RNA are highlighted in red. Non-native G nucleotides are highlighted in grey. E+F) CD melting curves of the 75mer (e) and the stabilized cold conformation of the 75mer (f) in 25 mM potassium phosphate buffer (pH 6.3) at a concentration of 10 µM. Melting points are marked with arrows.

To validate these structural models, we introduced mutations to selectively stabilize the cold conformation (U1815C and U1816C), which replace two GU base pairs with two more stable GC base pairs (Figure 6(c)). Furthermore, we devised a construct where we selectively stabilized the hot conformation by shortening the 75mer to a 50mer, removing the sequence elements that are involved in structural elements in the cold conformation, but are single-stranded in the hot conformation (Figure 6(d)).

The subsequent inline-probing analysis of the cold conformation mutant showed that it was indeed significantly stabilized by the mutations (Figure 6(a)). In contrast to the non-stabilized 75mer, only a slight increase in spontaneous cleavage of the RNA was detected with increasing temperature in the sequence regions nt1818-nt1830, nt1849-nt1854 and nt1863-nt1870 (cf. red boxes in Figures 6(a) and 5(c) or for direct comparison cf. Supplementary Figure S9). However, compared to the unstabilized 75mer, an increase in spontaneous cleavage with increasing temperature was detected in the loop region nt1837-1847 (cf. blue boxes in Figures 6(a) and 5(c) or for direct comparison cf. Supplementary Figure S9). We attribute this finding to weak base pairs between nucleotides within the loop, which are less stable when the temperature rises. CD analysis of the cold conformation showed a more defined transition and a slightly increased T_m_ (30°C versus 25°C for the WT) for the suggested cold stabilizing mutation (Figure 6(e, f)). This finding supports an overall stabilizing effect of the mutation at lower temperatures, even though the temperatures between CD and in-line probing can only be qualitatively compared due to differences in measurement conditions.

The in-line probing analysis of the stabilized hot conformation (Figure 6(b)) showed a temperature-dependent increase in spontaneous cleavage for the sequence ranges nt1862-1870 and nt1850-1854 (cf. red boxes). A similarly increased spontaneous cleavage of these sequence ranges could also be detected for the unstabilized 75mer at 45°C (cf. red boxes Figure 5(c)). Since there are no alternative cleavage patterns observed for the 50mer, this confirms the structural model for the hot conformation of the 75mer (Figure 5(d)).

In order to further confirm the temperature-dependent folding model of the 3ʹ pre-mRNA structure, we next examined both the 75mer and the stabilized hot conformation using NMR spectroscopy.

### NMR analysis of pre-mRNA structures at the 3ʹ splice site

The helix-bulge-stem loop structure of the stabilized hot conformation could partly be assigned by analysis of ^1^H,^1^H NOESY and ^1^H,^15^N TROSY spectra (Supplementary Figure S10A). Within the structure, imino proton resonances of the stem containing the GU base pair G1872-U1838 could be assigned (Supplementary Figure S10A, cf. red box in the second structure inset). To confirm the assignment, the basal stem region was also examined separately in the form of a 23mer using the same NMR experiments (Supplementary Figure S10B). This construct showed the same imino correlations that were observed for the stabilized hot conformation. Imino protons of the AU-rich stem containing A1860 and the small stem containing G1848 were not clearly detected and thus could not be assigned in the spectra of the hot conformation, presumably due to limited stability of this motif, which would result in decreased signal intensity due to rapid exchange with the solvent. However, an unassigned U and G imino proton signal could be detected in the canonical range of the TROSY spectrum (Supplementary Figure S10A). We tentatively assign this U imino proton signal to originate from the stem containing A1860, since it showed two cross peaks in the NOESY spectrum. We also assume that the G imino proton signal for which no NOESY crosspeaks could be detected is G1848. The signals of the unassigned U’s between 10.2–11.2 ppm very likely result from weak non-canonical base pairings in loop regions.

Interestingly, the structure which we denominate as the cold conformation is of such limited stability that neither the wildtype RNA nor the stabilized cold conformation construct of the 75mer generate NMR signals sufficient for an assignment, despite successful prediction of their secondary structures. An overview of the free energies of these constructs as well as the free energies of the hot conformation and the stabilized hot conformation at various temperatures determined with the RNAstructure folding software are given in Supplementary Table. S1.

**Figure 7.**
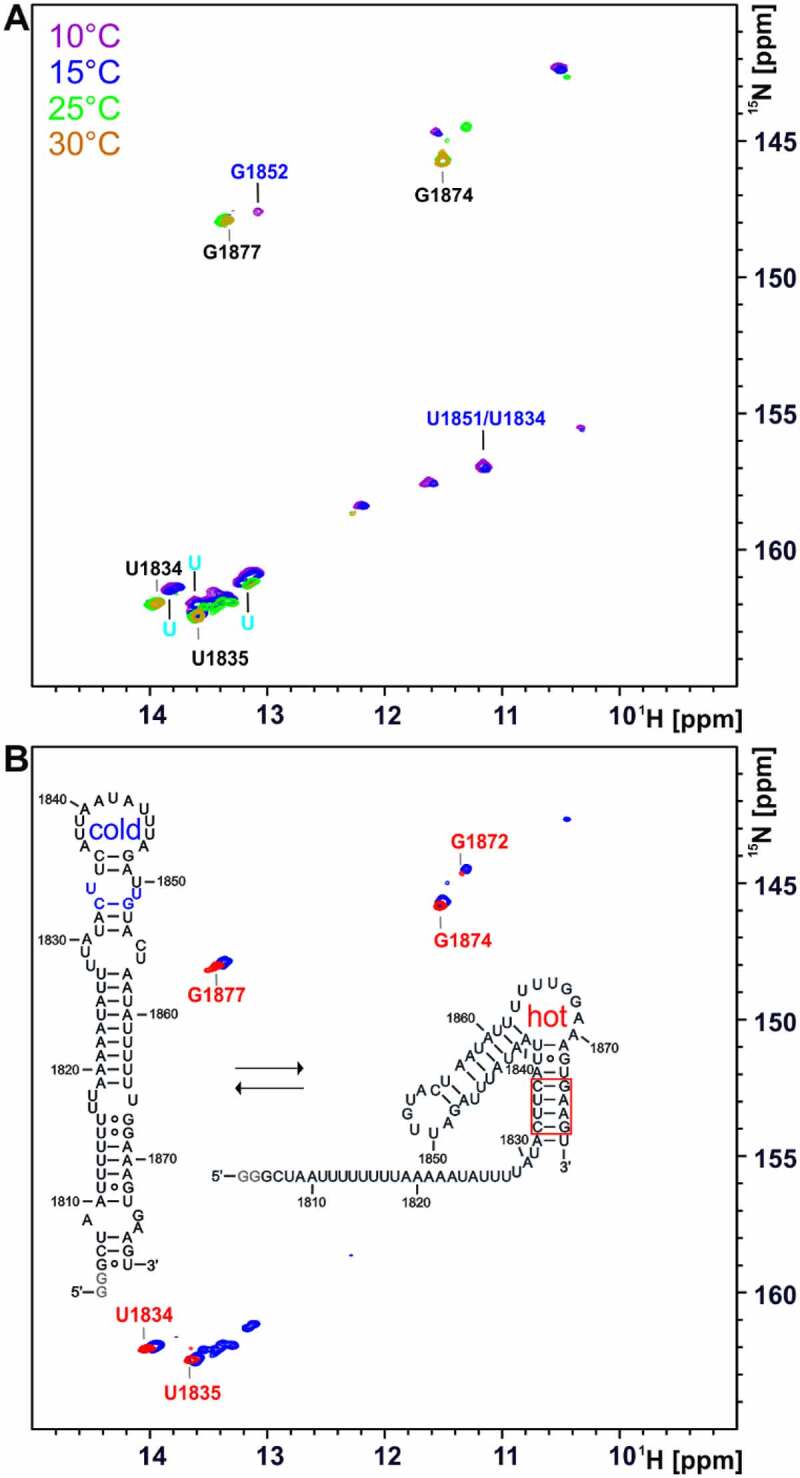
Temperature-dependent 2D NMR analysis of the 75mer located at the 3’SS of intron 2 of the HsfA2 pre-mRNA and comparison with its stabilized hot conformation. (a) ^1^H^15^N TROSY spectra of ^15^N GU-labelled 75mer (200 µM) at increasing temperatures (10°C-30°C). Spectra were recorded in 25 mM potassium phosphate buffer (pH 6.3) with 8% D_2_O at 600 MHz with 1024 × 256 points and 64 scans. Signals most likely originating from the hot conformation of the 75mer are labelled in black. Some signals that originate from the cold conformation of the 75mer RNA are labelled in light and dark blue. (b) Overlay of TROSY spectra of ^15^N GU-labelled 75mer (blue) and the ^15^N GU-labelled stabilized hot conformation of the 75mer (red) at 25°C in 25 mM potassium phosphate buffer (pH 6.3) with 8% D_2_O. Assigned signals of the stabilized hot conformation are labelled in red. The spectrum of the 75mer (200 µM) was recorded at 600 MHz with1024x256 points and 64 scans. The spectrum of the stabilized hot conformation (30 µM) was recorded at 950 MHz with 1024 × 256 points and 128 scans.

To characterize the temperature-dependent change of the conformation of the 75mer RNA, ^1^H,^15^N TROSY spectra were recorded at different temperatures (Figure 7(a)).

The spectra of the 75mer showed several signals with increased intensity at elevated temperatures (at a ^1^H chemical shift of ~14 ppm, ~13.6 ppm, ~13.4 ppm and ~11.5 ppm, intense orange signals). The overlay of the TROSY spectra of the 75mer RNA and the stabilized hot conformation at 25°C showed that these signals most likely originate from the hot conformation of the RNA due to the similar chemical shift (Figure 7(b), the signals of the stabilized hot conformation are marked in red). The assignment of the stabilized hot conformation was therefore transferred to the spectra of the 75mer RNA (Figure 7(a), labelled in black). At 30°C, almost exclusively imino proton signals of the suspected hot conformation were detectable in the 75mer TROSY spectrum (Figure 7(a), labelled in black). Some NMR signals of the cold conformation of the 75mer were determined by the analysis of a 48mer, which is a shortened and stabilized version of the cold conformation of the 75mer (Figure 7(a), coloured light/dark blue and Supplementary Figure S11). Furthermore, NOESY spectra of the 75mer RNA were recorded in a temperature range of 0–30°C and an assignment attempt was made (Supplementary Figures S11 and S12).

**Figure 8.**
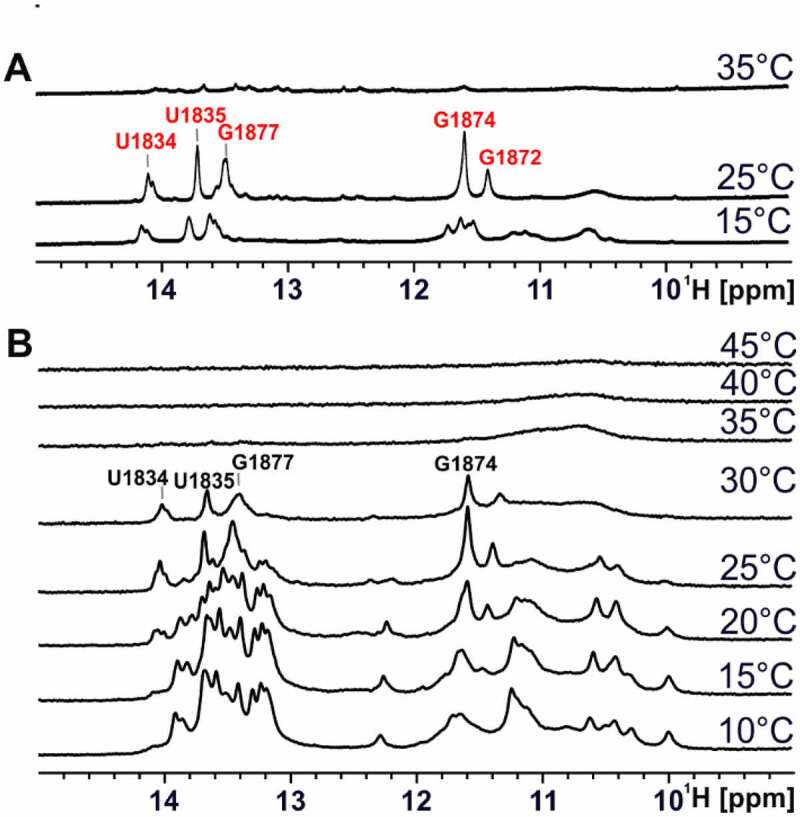
Temperature-dependent 1D NMR spectra of the stabilized hot conformation and 75mer. (a) Imino proton region of 1D ^1^H spectra of the stabilized hot conformation of 75mer (80 µM) at increasing temperatures (15°C-35°C) in 25 mM potassium phosphate buffer (pH 6.3) with 8% D_2_O at 950 MHz with 1536 scans. Assigned signals of the stabilized hot conformation are labelled in red. (b) Imino proton region of 1D ^1^H spectra of 75mer (200 µM) at increasing temperatures (10°C-45°C) in 25 mM potassium phosphate buffer (pH 6.3) with 8% D_2_O at 600 MHz with 512 scans. Signals most likely originating from the hot conformation of the 75mer are labelled in black.

We then recorded ^1^H 1D imino proton spectra of the 75mer RNA at various temperatures between 10–45°C (Figure 8(b)). While the 1D spectra showed only subtle differences in the temperature range between 10°C and 20°C, significant differences in the signal intensity ratios become visible at temperatures above 25°C, pointing to a cooperative folding transition of the RNA into the hot conformation. In the temperature range between 35°C and 45°C, no clear imino proton signals could be detected. We performed the same type of analysis for the stabilized hot conformation (Figure 8(a)). Here, differences in the signal intensity ratios were detected for the spectra at 15°C and 25°C. At 35°C, no intensity corresponding to imino proton signals could be detected. From comparison of spectra from the 75mer at 25°C or 30°C with the stabilized hot conformation at 30°C, it becomes obvious that these are almost identical, which again strongly suggests that the 75mer changes from the cold conformation to the hot conformation upon an increase in temperature.

It is important to note that, according to the structure prediction of RNAstructure [17], the refolding process of this 75mer pre-mRNA fragment from HsfA2 *S. lycopersicum* is identical in many other tomato species. Especially the sequence regions of the assigned stem of the hot conformation is 100% conserved (Supplementary Figure S13).

In summary, the data collected from the pre-mRNA fragments of intron 2 of HsfA2 from *S. lycopersicum* at the 3ʹ splice site show that the RNAs are thermally rather unstable, and undergo distinct conformational changes in contrast to the fragments at the 5ʹ splice sites of this intron.

## Discussion

In this work we describe pre-mRNA structures at the 3ʹ splice site and the 5ʹ splice sites of intron 2 of the heat shock factor HsfA2 from the tomato plant *S. lycopersicum*. We investigate whether these pre-mRNA fragments can regulate the temperature-dependent alternative splicing of this intron [9]. A possible regulation mechanism for these fragments would be if they, acting as RNA thermometers, influence the accessibility of splice sites through switch-like conformational changes. Furthermore, we structurally compare pre-mRNA fragments of the species *S. lycopersicum* and *S. peruvianum* at the 5ʹ splice site of the intron to analyse the influence of SNPs on the pre-mRNA structure, as it was shown that three specific SNPs (GGG/AAA) influence the splicing efficiency [9].

The analysis of the pre-mRNA fragments at the 5ʹ splice site shows that any reported temperature dependence in alternative splicing is not correlated to temperature-dependent structural switches between different conformations of the pre-mRNA fragments. Hu et al. showed for intron 2 of the HsfA2 pre-mRNA of *S. lycopersicum* that the first 5ʹ splice site is used in preference to the second 5ʹ splice site at low temperatures (30°C) and that this is reversed when the temperature increases (42°C) [9]. We were able to show that these three SNPs lead to structural differences in the pre-mRNA structure of the species. The different splicing efficiency, which is determined by the more or less frequent use of the first 5ʹ splice site is not induced by different structural preferences of the pre-mRNA, as the first splice site in both species is located in an identical and equally stable structural area. Our analysis of the 5ʹ splice sites for both *S. lycopersicum* and *S. peruvianum*, which differ by three G-to-A SNPs, show that the intron adopts different secondary structures, as evidenced by both in-line probing and NMR. Both these structures show a higher degree of thermal stability, suggesting that on their own those structures would not be able to mediate a regulatory change in splicing.

Regarding the biological function, the study by Hu et al. [9] shows that intron 2 of HsfA2 of *S. lycopersicum* is utilized less with increasing temperature. This presumably is due to a decreased use of the 3ʹ splice site (and consequently also less use of the 5ʹ splice sites) in a temperature-dependent fashion. Our analysis of the RNA fragments at the 3ʹ splice site by both in-line probing and NMR spectroscopy show an intronic pre-mRNA fragment that ‘switches’ its conformation when the temperature changes. The temperature-dependent structural plasticity of the pre-mRNA is remarkable. We identify and structurally describe the conformations that are characteristic for ‘cold’ and ‘hot’. Despite environmental or measurement differences between NMR spectroscopy, in-line-probing and the *in vivo* situation where alternative splicing has been characterized, this structural switch and its transition temperature is compatible with its possible role in alternative splicing. At higher temperatures, this splice site is less utilized, despite its slightly increased accessibility shown in our data. Surprisingly though, a similar 3ʹ splice site sequence is also present in other species such as *S. peruvianum*. There are two plausible models how the temperature-dependent conformational change at the 3’SS would result in alternatively spliced intron 2 of HsfA2. First, a long-range interaction between any region of the 5ʹ splice site and exclusively one conformation of the thermosensitive 3’SS could determine retention of this intron. Second, an interaction of a splicing factor with again selectively one of the conformations of the 3’splice site could result in alternative splicing. While we cannot substantiate either one of those models, there is precedent for at least the former model [14].

In theory, an RNA splicing temperature-dependent riboswitch would regulate the temperature-dependent alternative splicing of an intron if it changes its conformation like a ‘switch’, blocking specific regulatory sites (including splicing silencers and/or enhancers [18]) while simultaneously exposing others. Based on our analysis of the pre-mRNA fragments at the splice sites, these appear to not function as such RNA thermometers in the absence of associated proteins. As shown by in-line probing, neither the 3’SS nor the 5’SS are accessible by themselves, but likely require additional splicing factors [19]. Identification of protein splice factors has remained enigmatic until now.

**Figure 9.**
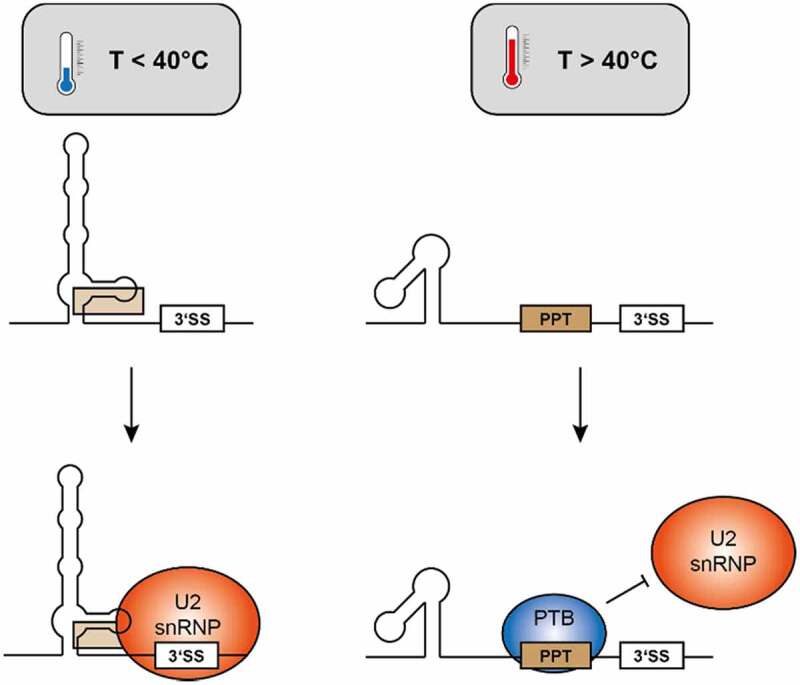
A potential model for a molecular mechanism governing alternative splicing of the 3ʹ splice site of intron 2 of HsfA2. Left: At temperatures below 40°C, an extended secondary structure encompassing the region 3ʹ of the 3’SS is formed, allowing initiation of splicing by binding of the U2 snRNP. Right: At higher temperatures, a highly stable, alternative structure is formed, rendering a potential PPT single-stranded to allow for binding of regulatory proteins, blocking binding of the U2 snRNP.

The intronic sequence elements surrounding the 3’SS are a likely polypyrimidine tract (nt ~1890 through 1910), followed by the 3ʹ element that folds in a temperature dependent fashion (Figure 5). Since the exact location of the branchpoint adenosine is unknown [20], it is possible that the temperature-dependent structure encompasses the branchpoint A. The alternative, temperature-dependent conformations would (among others) support the following model (Figure 9):

At higher temperatures, the hairpin-bulge-hairpin structure (nts 1833–1878) which is selectively stabilized at higher temperatures, may provide a stable folding platform, presenting a single-stranded polypyrimidine tract (PPT) between this element and the 3’SS. Potential binding of a PTB that suppresses splicing at the 3’SS would then result in intron retention.

At lower temperatures, binding of this PTB would be reduced or eliminated, since the PPT is engaged in interactions with 3ʹ sequence elements, and would thus be not available in a single-stranded, protein-binding competent conformation. In absence of protein binding to the PPT, the U2 snRNP would be able to bind to the 3’SS, and splicing can occur. Here again, it is worth noting that the 3’SS is only used at low temperatures (~< 40°C) and no longer at high temperatures (~˃ 40°C) (Figure 1).

Our data show that such splice factors are likely required for differential splicing: While the thermally more stable 5’SS remains spliced even at elevated temperatures, the unavailability of the 3’SS at higher temperatures is not due to a lack of accessibility, but rather linked to an overall instability exactly around the 3’SS. The 3’SS is additionally involved in alternative conformations, whose population is differently affected by an increase of temperature. In order to conduct pulldown experiments, an enrichment of the substantially different conformation at higher temperature provides one suggestion derived from our experiments for future research. For the pre-fragments at the 5ʹ splice sites of *S. peruvianum* and *S. lycopersicum*, we show that – independent of the temperature – they assume distinct conformations which each have a relatively high melting point, likely beyond the temperature range for which alternative splicing was detected. Three SNPs are responsible for the difference in structure, which on its own hints at the requirement for additional proteins. Another possible explanation is that formation of the highly stable structure at higher temperatures prevents other interactions between sequence motifs, which could sequester intronic or exonic splicing silencers or engage splicing enhancers, which again may depent on the interactions with other RNA-binding proteins.

Our data thus delineate the possible role of RNA structural changes: regulation could thus come about by RNA-protein complexes that exhibit selective binding to different RNA conformations. Stabilization of either one of the two conformations for identification of potential protein binding to the pre-mRNA could help identify such temperature-dependent splice modulating proteins in plants.

## Material and methods

### Transcription template

Transcription templates were prepared by polymerase-chain reaction. Transcription templates for 74mer*^S.lyco^*, 74mer*^S.peruv^*, 37mer*^S.lyco^*, 59mer*^S.lyco^* and the 143mer were obtained by using the corresponding PCR template of the 153mer and varying the primers. The PCR template of 75mer and the stabilized hot conformation (47mer, used for preparation of the NMR sample) and all primers were purchased from Eurofins Genomics. Transcription templates for the stabilized cold conformation and stabilized hot conformation (50mer, used for preparation of the RNA for inline-probing) were obtained by using the PCR template of the 75mer and varying the primers. PCR was performed in reaction volumes of 100 µL with 0,5 µM of each primer, 200 µM of each dNTP, 200 ng DNA template and Phusion® High-Fidelity DNA Polymerase according to standard protocols from NEB (Supplementary Table S1 and S2). The transcription template for 18mer*^S.peruv^* was generated by annealing the two DNA oligonucleotides 5ʹ-GATCTCTAAAGGAAACCCTATAGTGAGTCGTATTA*-3ʹ* (the underlined region corresponds to the inverse complementary T7 promoter sequence) and 5ʹ-TAATACGACTCACTATAGGG-3ʹ (T7 promotor) at a concentration of 100 µM of each DNA at 95°C followed by cooling the mixture to 25°C. The oligonucleotides were purchased from Eurofins Genomics.

### RNA preparation

All RNAs studied in this work, except for the 23mer (purchased from Dharmacon (Lafayette, USA)), were synthesized by *in vitro* transcription with T7 RNA polymerase from PCR products or DNA template (Supplementary Table S3 and S4). All transcriptions were optimized for Mg^2+^, NTPs and DMSO concentrations to achieve maximum yields and homogeneity of the transcripts. For NMR experiments 5–20 mL transcription reactions were performed and for in-line probing experiments 500 µL transcriptions were performed. Transcriptions were incubated for 16 h at 37°C in transcription buffer (200 mM TrisHCl; pH 8,1) with 2 mM spermidine, 5–7,5 mM of each NTP, 10–80 mM Mg(OAc)_2_, 20 mM dithiothreitol (DTT), 20% (v/v) of DMSO, 4–6% (v/v) of the PCR mixture or 0,4 µM DNA template and 150 nM T7 RNA polymerase (homemade). Unlabelled NTPs were purchased from Carl Roth GmbH (Karlsruhe). ^15^N-labelled GTP and UTP were purchased from Silantes (Munich).

RNAs for NMR experiments were desalted with 160 mL ddH_2_O using centrifugal concentrators with a molecular weight cut-off of 3000 (Vivaspin 20 from Sartorius AG, Goettingen) and purified by denaturing urea PAGE (10–20% 29:1 (w/w) acrylamide/bisacrylamide, 7 M urea). The RNAs were visualized by UV shadowing (254 nm), excised from the gel, and eluted with ddH_2_O at 4°C for 10–34 h. Precipitation was performed with 0,6 M sodium acetate (pH 5,5) and four volumes of ethanol (−20°C, 16 h). The RNAs were folded by 7 min denaturation at 85°C at a concentration of 50–350 µM and immediate dilution to 5–35 µM with water followed by cooling to room temperature and incubation on ice for 30 min. Afterwards the RNAs were exchanged into NMR buffer (25 mM K_2_HPO_4_/KH_2_PO4, pH 6,3) using centrifugal concentrators with a molecular weight cut-off of 3000 (Vivaspin 20 from Sartorius AG, Goettingen). NMR samples were prepared with 8% D_2_O and 50 µM DSS as chemical shift standard.

RNAs for in-line probing experiments were desalted and buffer exchanged after transcription using gel filtration columns (illustra NAP-5 Columns, GE Healthcare). The RNAs were precipitated with 0,5 M ammonium acetate and 2,5 volumes of ethanol (−20°C, 16 h). 5 nmol of RNA were dephosphorylated in 1x CutSmart® Buffer (50 mM potassium acetate, 20 mM tris-acetate, 10 mM magnesium acetate, 100 μg/ml BSA, pH 7.9, New England Biolabs) with 10 units Shrimp Alkaline Phosphatase (New England Biolabs) at 37°C for 5 h in a reaction volume of 100 µL and purified by denaturing urea PAGE (8–10% 29:1 (w/w) acrylamide/bisacrylamide, 7 M urea). The RNAs were visualized by UV shadowing (254 nm), excised from the gel, and eluted with ddH_2_O at 4°C for 10–34 h. The RNAs were precipitated with 0,6 M sodium acetate (pH 5,5) and four volumes of ethanol (−20°C, 16 h). For radiolabeling the 5‘-termini ~60 pmol of dephoshorylated RNA was incubated at 37°C for 40 min with 10 units T4 Polynucleotide Kinase (Thermo Scientific) and ~10 pmol γ-^32^P-ATP in 1x reaction buffer A for T4 Polynucleotide Kinase (Thermo Scientific) in a reaction volume of 30 µL. The radiolabeled RNA was purified by denaturing urea PAGE (8% 29:1 (w/w) acrylamide/bisacrylamide, 7 M urea), visualized using a phosphor imager (Storage Phosphor Screen, GE Healthcare) and a scanner (Typhoon FLA 9500, GE Healthcare), excised from the gel, and eluted and precipitated as described above.

### RNA secondary structures

All secondary structures drawn were generated either by Mfold [21] or RNAstructure [22].

### In-line probing

Temperature dependent in-line probing was performed with ~1 pmol of 5‘ ^32^P-labelled RNA in reaction buffer (50 mM Tris-HCl; 20 mM MgCl_2_; 100 mM KCl, pH 8,3) for 1,5–58 h in a temperature range of 10–45°C in a reaction volume of 20 µL. Prior to reaction the RNA ~1 pmol was unfolded in 10 µL ddH_2_O at 85°C for 5 min followed by cooling to room temperature. The reaction times were optimized respective to probing temperature. The reaction time at 45°C was 1–1,5 h, at 35°C 3,5 h and at 10–20°C 48–58 h. Reactions were terminated by the addition of an equal volume of gel loading buffer containing 10 M urea and 25 mM EDTA.

RNA cleavage ladders were generated by incubating ~1 pmol of 5‘ ^32^P-labelled RNA in cleavage solution (50 mM NaHCO_3_/Na_2_CO_3_, pH 9) for 5–10 min at 95°C in a reaction volume of 10 µL. G-specific sequencing ladders were generated by incubating ~1 pmol of 5‘ ^32^P-labelled RNA with 0,5 U RNase T1 in G-buffer (6,25 mM sodium citrate (pH 4,5); 1,75 M Urea; 0,25 mM EDTA) for 10–25 min at 55°C in a reaction volume of 10 µL. Reactions were quenched with an equal volume of gel loading buffer and cooling to −20°C.

The reaction products were analysed by denaturing urea PAGE (8–12% 29:1 (w/w) acrylamide/bisacrylamide, 7 M urea) and visualized using a phosphor imager (Storage Phosphor Screen, GE Healthcare) and a scanner (Typhoon FLA 9500, GE Healthcare).

### NMR spectroscopy

NMR experiments were conducted on 600 MHz, 700 MHz, 800 MHz and 950 MHz Bruker spectrometers equipped with 5 mm ^1^H (^13^C/^15^N) cryogenic probes and z-axis gradient systems. Temperature dependent 1D ^1^H-NMR spectra were recorded with a jump-return echo experiment [23] in a temperature range of 10–45°C. 2D [^1^H,^15^N]-TROSY [24,25] and 2D [^1^H,^15^N]-SOFAST-HMQC [26] spectra were recorded in a temperature range of 10–30°C. [^1^H,^1^H]-NOESY spectra were recorded with a jump-return echo water suppression technique [23] at 10°C and 30°C.

### CD spectroscopy

CD melting curves were measured on a JASCO spectropolarimeter J-810 with a temperature slope of 1°C/min at a wavelength of 262 nm. The samples were measured at a concentration of 10 µM in NMR buffer (25 mM K_2_HPO_4_/KH_2_PO_4_, pH 6,3) in a cuvette with a sample diameter of 1 mm. For melting point analysis, the temperature was plotted against the folded fraction of the RNA as described [27]. The melting points of 74mer^*S.peruv*^, 37mer^*S.lyco*^ and the 75mers were determined using a Boltzmann Fit in OriginPro 8. For 74mer^*S.lyco*^, the melting point was determined with a Double Boltzmann Fit in Origin 2021b.

## Supplementary Material

Supplemental Material
