# Supplementary material for: Structural analysis of temperature-dependent alternative splicing of HsfA2 pre-mRNA from tomato plants
Source: RNA Biol. 2022 Feb 7;19(1):266–78. doi: 10.1080/15476286.2021.2024034 (PMC8824230; doi:10.1080/15476286.2021.2024034)
Supplement: Supplemental Material [file KRNB_A_2024034_SM3394.zip › supplementary/suppl info.pdf]

Supplementary Information:

Structural analysis of temperature-dependent alternative splicing of HsfA2 pre-mRNA from tomato plants

Patrizia Broft[1], Remus Rosenkranz[2], Enrico Schleiff[2], Martin Hengesbach[1]*, Harald Schwalbe[1]*

Supplementary Figure S1:

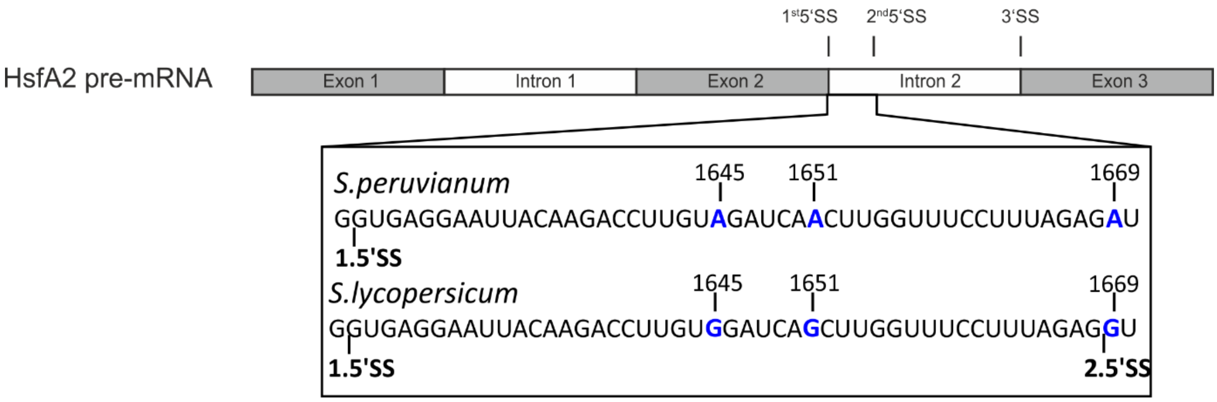

Sequence section of the HsfA2 pre-mRNA from the plant species *S. peruvianum* and *S. lycopersicum* and the SNPs occurring therein. The non consecutive SNPs (3A's/3G's) are highlighted in blue in the sequence. The SNPs are decisive for the different splicing efficiency of intron 2 of the two plant species.

Supplementary Figure S2:

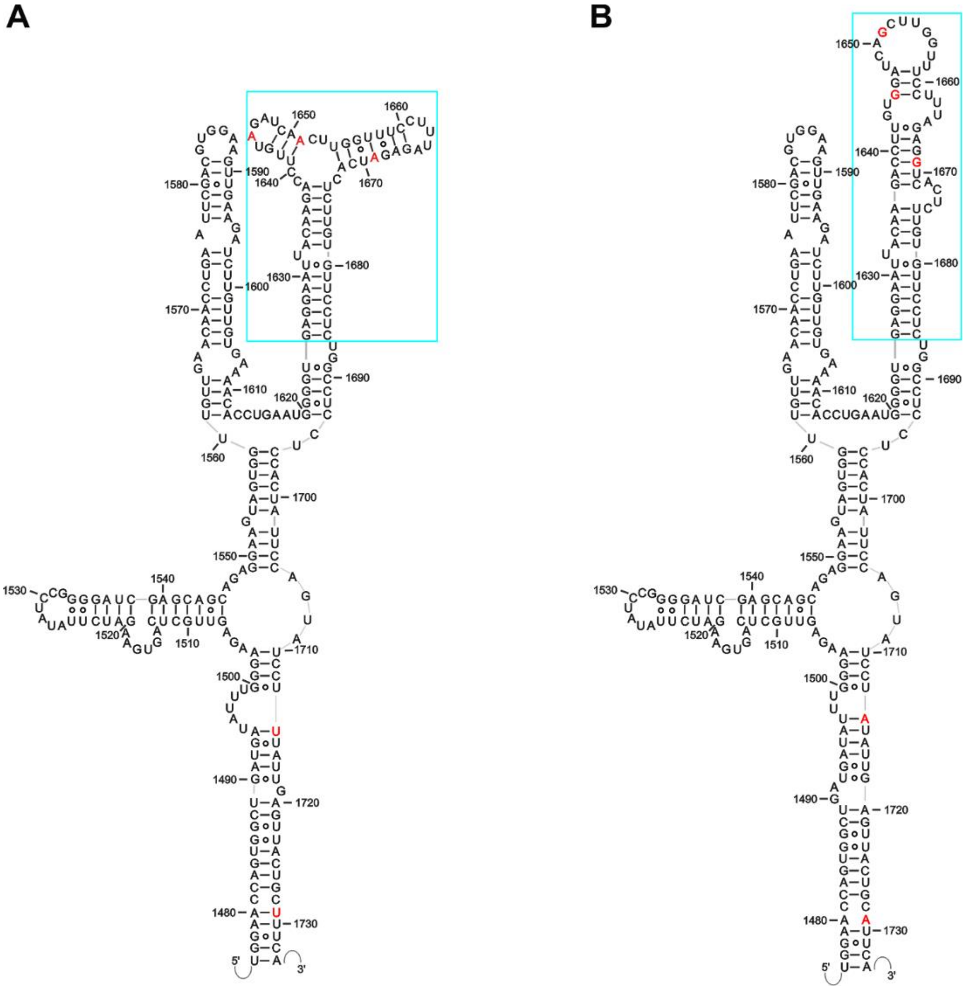

Mfold structure prediction of the large HsfA2 pre-mRNA fragment (617nt; nt1439-nt2055) of *S. peruvianum* and *S. lycoperiscum*. The fragments of of *S. peruvianum* (A) and *S. lycoperiscum* (B) were used for construct design at the 5'SS. SNP's are highlighted in red and and missing sequence areas are abbreviated with dark gray curved lines. The structural area of the Mfold structure prediction that matches to the structural model of the 153mer$^{S.lyco}$ and 153mer$^{S.peruv}$ is marked with a turquoise box.

Supplementary Figure S3:

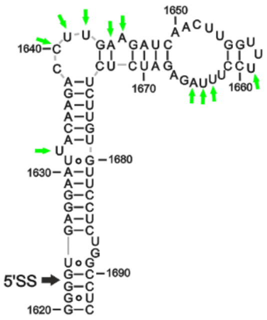

74mer
S.peruvianum

Alternative structural model of 74mer$^{S.peruv}$ derived from analysis of in-line probing, and including Mfold prediction. The splice site is marked with an arrow. Nucleotide positions at which a relatively large amount of spontaneous cleavage was found in the in-line probing experiment are marked with green arrows.

Supplementary Figure S4:

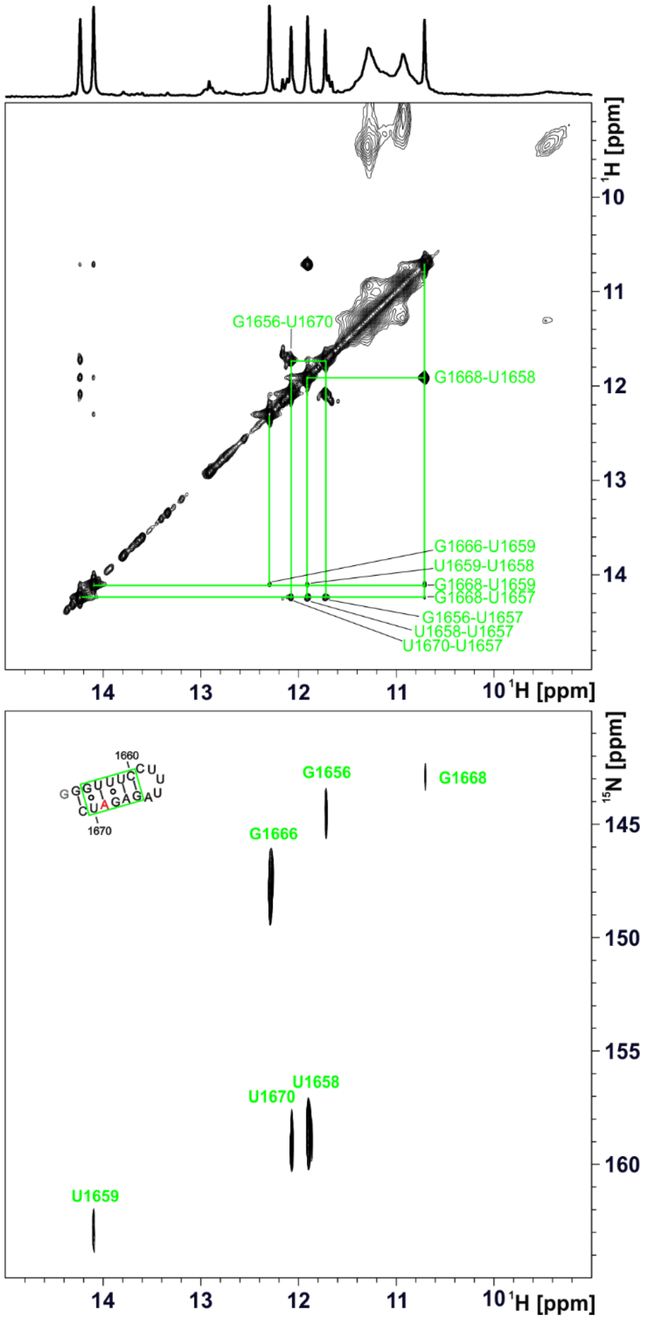

NMR analysis of 18mer$^{S.peruv}$. ^{1}H,^{1}H-NOESY and ^{1}H,^{15}N-SFHMQC spectrum of 18mer$^{S.peruv}$ (nt1655-nt1671 of the pre-mRNA) annotated with assignment. Imino-proton correlations between consecutive base pairs (imino walks) are shown in green in the NOESY spectrum. The secondary structure determined experimentally is shown in the inlet (non native G is highlighted in grey). Structural sections that could be assigned are marked with a box in the secondary structure. The spectra of the RNA were recorded in 25 mM potassium phosphate buffer (pH 6.3) with 8% D_2O at 10°C. Top: NOESY spectrum of 18mer$^{S.peruv}$ (~500 µM) recorderded at 800 MHz with 2048 × 640 points and 224 scans. Bottom: SFHMQC spectrum of 18mer$^{S.peruv}$ (~500 µM) at 800 MHz with 2048x640 points and 224 scans. This sample could not be completely purified by means of gel purification, for this reason additional signals are visible in the NOESY spectrum.

Supplementary Figure S5:

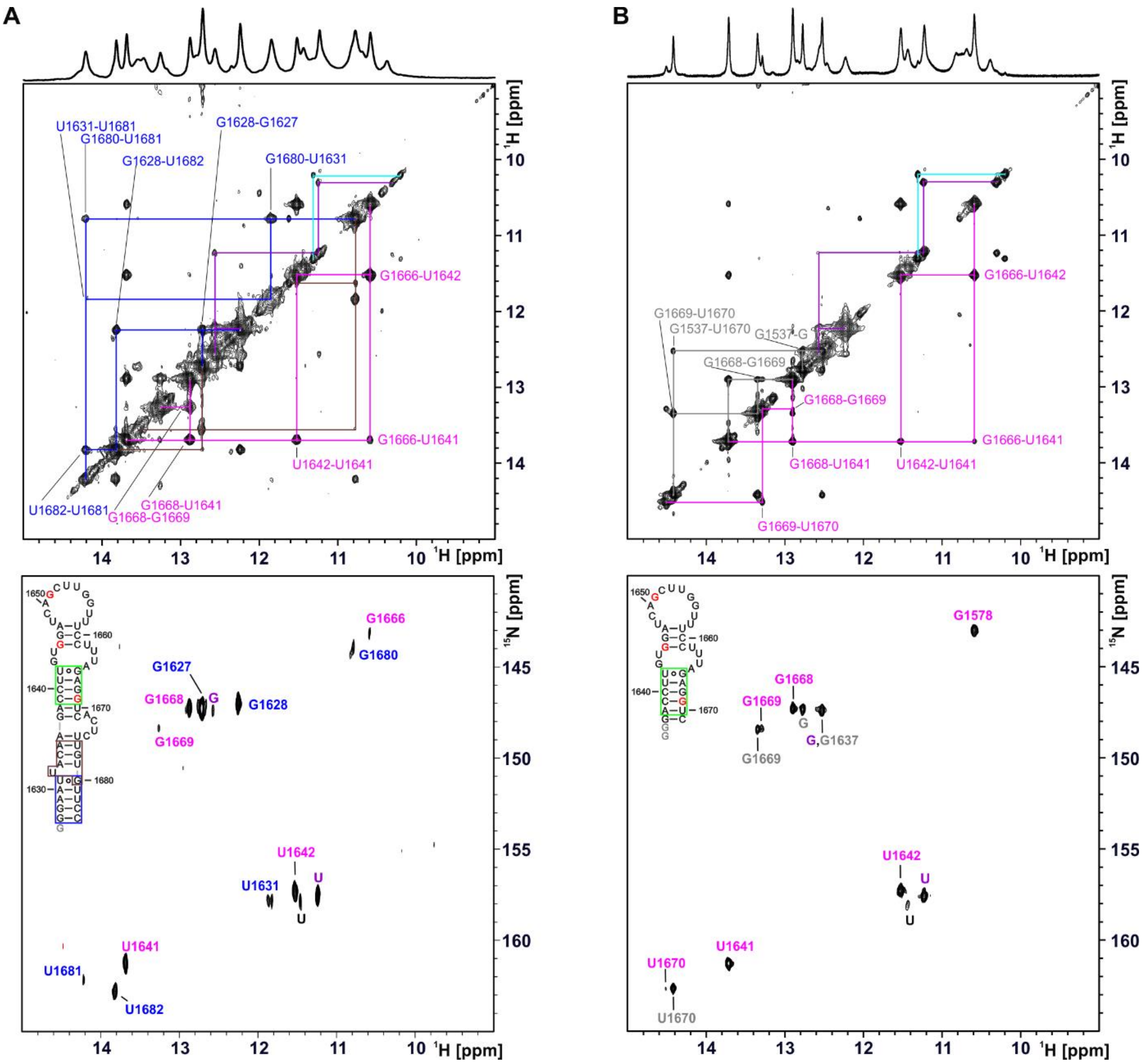

NMR analysis of 59mer$^{S.lyco}$ and 37mer$^{S.lyco}$. **A+B)** ^{1}H,^{1}H-NOESY and ^{1}H,^{15}N-SFHMQC spectrum of 59mer$^{S.lyco}$ (nt1627-1684 of the pre-mRNA) (A) and 37mer$^{S.lyco}$ (nt1637-1671 of the pre-mRNA) (B) annotated with assignment. Imino-proton correlations between consecutive base pairs (imino walks) are shown in different colours in the NOESY spectra. The secondary structures determined experimentally are shown in the inlet. Structural sections that could be assigned are marked with boxes in the secondary structure with the same colour code that was used for the respective assignment. The spectra of the RNAs were recorded in 25 mM potassium phosphate buffer (pH 6.3) with 8% D_2O at 10°C. **A)** Top: NOESY spectrum of 59mer$^{S.lyco}$ (660 µM) was recorded at 800 MHz with 2048 × 512 points and 192 scans. Bottom: SFHMQC spectrum of 59mer$^{S.lyco}$ (660 µM) at 600 MHz with 2048x76 points and 2160 scans. **B)** Top: NOESY spectrum of 37mer$^{S.lyco}$ (1 mM) recorderded at 800 MHz with 2048 × 128 points and 126 scans. Bottom: SFHMQC spectrum 37mer$^{S.lyco}$ (1 mM) at 600 MHz with 2048x128 points and 2700 scans. Additional signals are due to inhomogenity of the sample at 3‘/5‘ end. Most of these signals are assigned in grey.

Supplementary Figure S6:

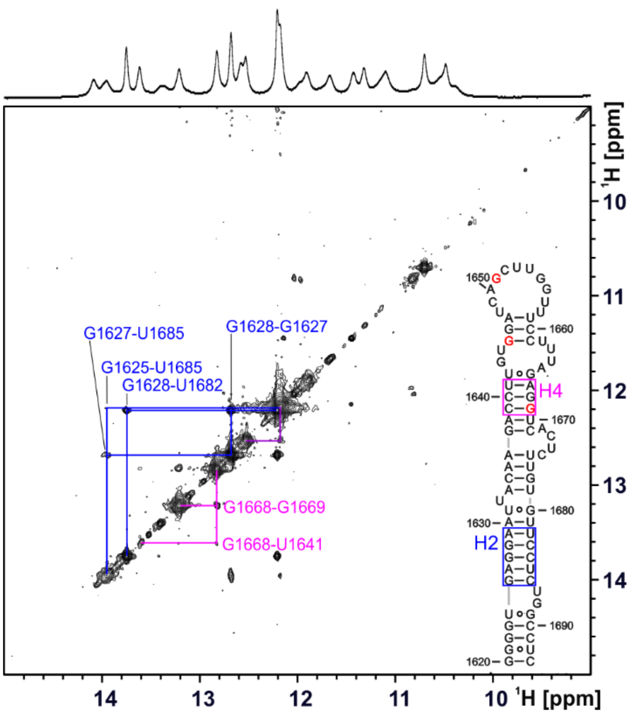

NMR analysis of 74mer$^{S.lyco}$ at 30°C. ^{1}H,^{1}H-NOESY spectrum at 30°C of 74mer$^{S.lyco}$ (930 µM) annotated with assignment. Imino-proton correlations between consecutive base pairs (imino walks) are shown in different colours in the NOESY spectrum. The secondary structure determined experimentally is shown in the inlet. Structural sections that could be assigned are marked with boxes in the secondary structure. The spectrum of the RNA was recorded in 25 mM potassium phosphate buffer (pH 6.3) with 8% D_2O at 800 MHz with 2048 × 992 points and 128 scans.

Supplementary Figure S7:

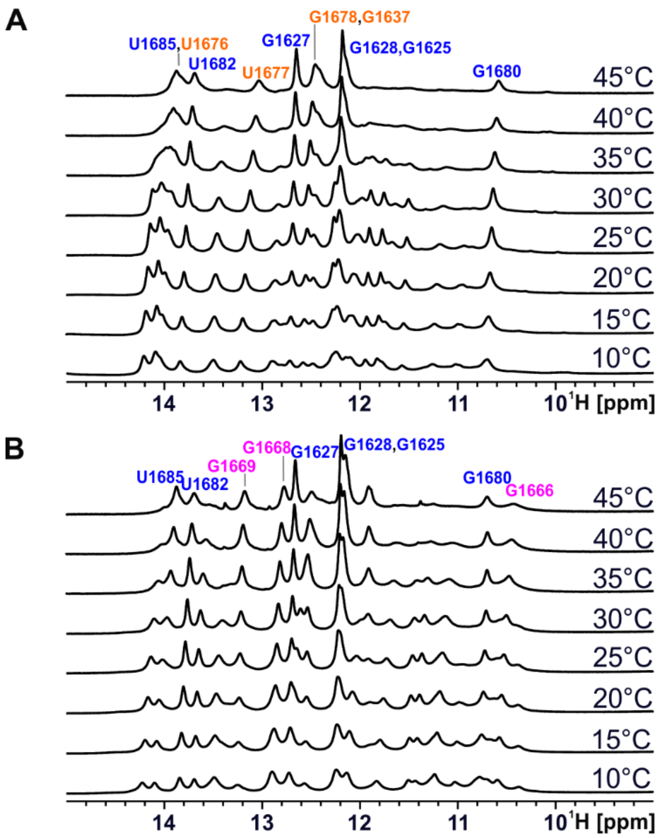

Temperature-dependent 1D NMR spectra of 74mer$^{S.peruv}$ and 74mer$^{S.lyco}$. **A+B)** Imino proton region of 1D ^{1}H spectra of 74mer$^{S.peruv}$ (A) and 74mer$^{S.lyco}$ (B) at increasing temperatures (10°C-45°C). Spectra were recorded in 25 mM potassium phosphate buffer (pH 6.3) with 8% D_2O at 600 MHz with 256 scans. The concentration of 74mer$^{S.peruv}$ was 1.2 mM and the concentration of 74mer$^{S.lyco}$ was 930 µM.

Supplementary Figure S8:

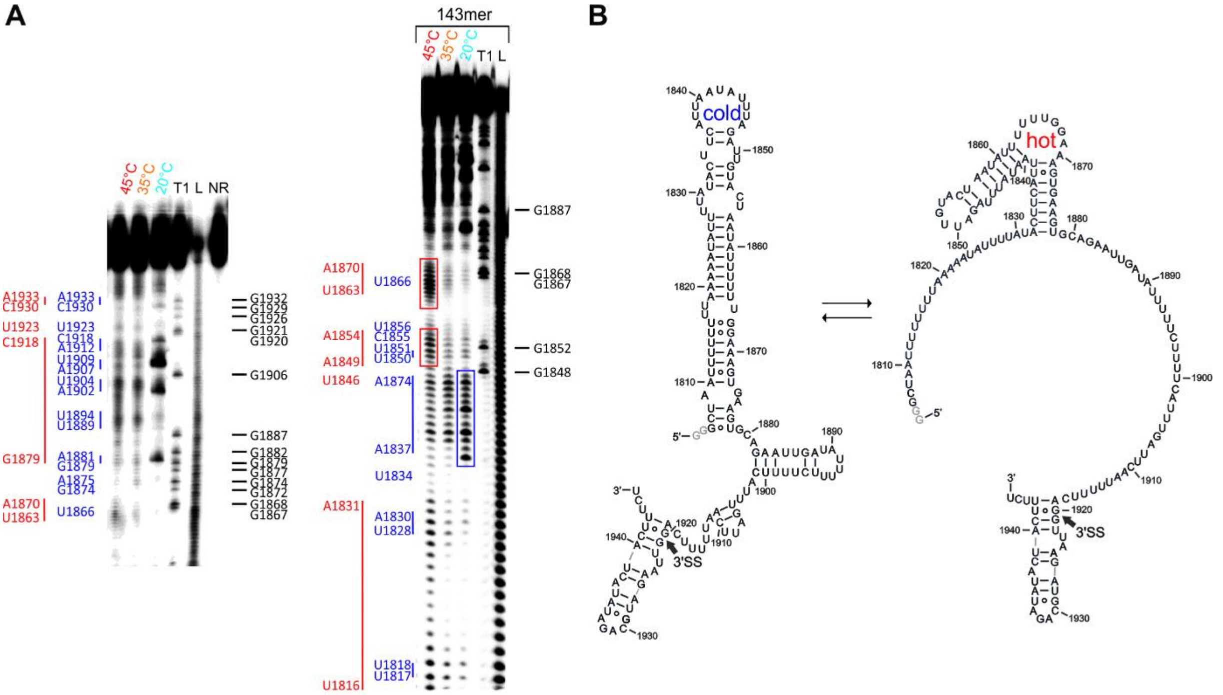

Temperature-dependent in-line probing analysis and structural models of 143mer$^{S.lyco}$ at the 3'SS. **A)** In-line probing analysis of 143mer$^{S.lyco}$ in a temperature range of 20-45°C. Lanes designated NR, T1, and L identify RNA samples loaded after subjecting to no reaction, partial digestion with RNase T1, or partial digestion with alkali, respectively. Bands corresponding to RNase T1 cleavage after G residues are assigned on the right side of each gel. Nucleotides or nucleotide sequences that are not base-paired in the structural model are assigned to the left side of the gel in blue for the cold conformation and in red for the hot conformation. **B)** Structure models of 143mer$^{S.lyco}$. The splice site (3'SS) is marked with an arrow and non-native G nucleotides are highlighted in grey.

Supplementary Figure S9:

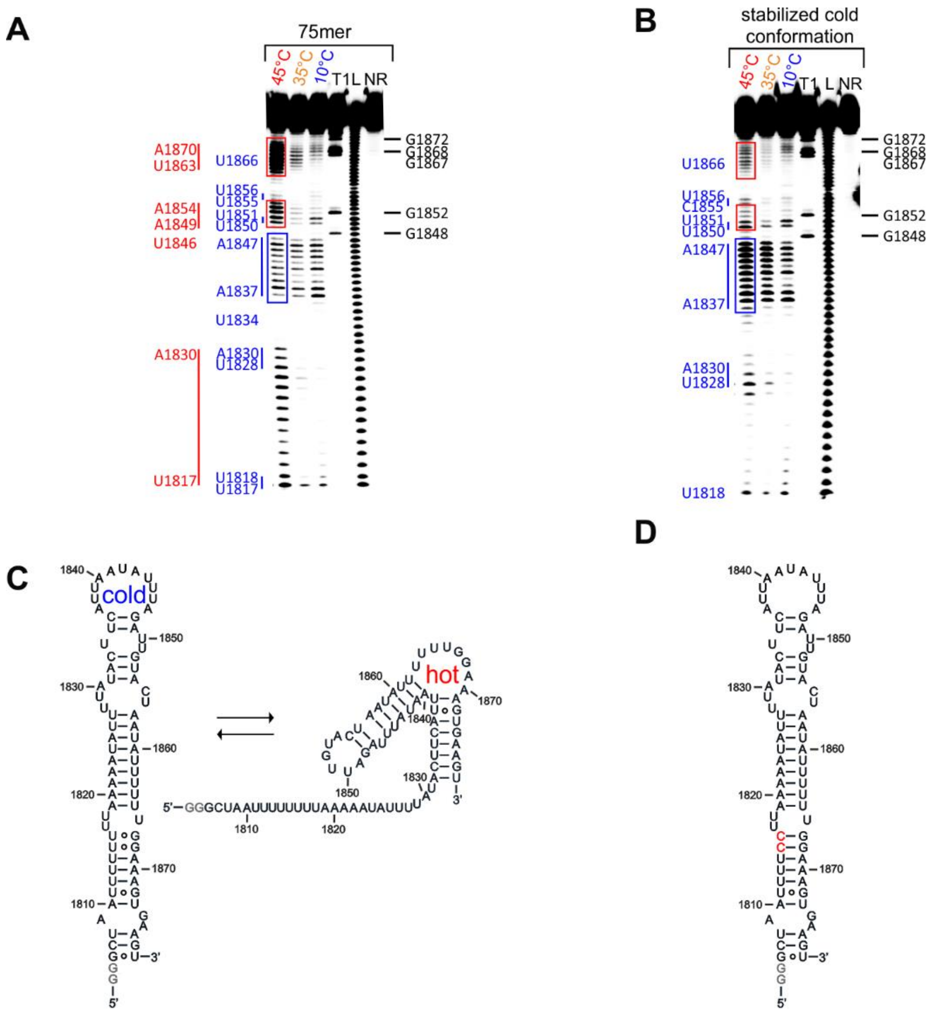

In-line probing analysis and structure models of 75mer and its stabilized cold conformation. **A)** In-line probing analysis of 75mer in a temperature range of 10-45°C. Lanes designated NR, T1, and L identify RNA samples loaded after subjecting to no reaction, partial digestion with RNase T1, or partial digestion with alkali, respectively. Bands corresponding to RNase T1 cleavage after G residues are assigned on the right side of the gel. Nucleotides or nucleotide sequences that are not base-paired in the structural model (C) are assigned to the left side of the gel in blue for the cold conformation and in red for the hot conformation. Intense bands of the hot conformation are marked with a red boxes. Prominent bands of the cold conformation are marked with a blue box. **B)** In-line probing analysis of the stabilized cold conformation of 75mer in a temperature range of 10-45°C. Lanes designated NR, T1, and L identify RNA samples loaded after subjecting to no reaction, partial digestion with RNase T1, or partial digestion with alkali, respectively. Bands corresponding to RNase T1 cleavage after G residues are assigned on the right side of each gel. Nucleotides or nucleotide sequences that are not base-paired in the structural model (D) are assigned to the left side of the gel. The position of prominent bands of the hot conformation are marked with red boxes. Prominent bands of the cold conformation are marked with a blue box. **C)** Structure model of the cold conformation (left) and hot conformation (right) of 75mer. Non-native G nucleotides are highlighted in grey. **D)** Structure model of the stabilized cold conformation of 75mer. The stabilizing mutations (2xC) of the cold conformation of the 75mer RNA are highlighted in red. Non-native G nucleotides are highlighted in grey.

Supplementary Figure S10:

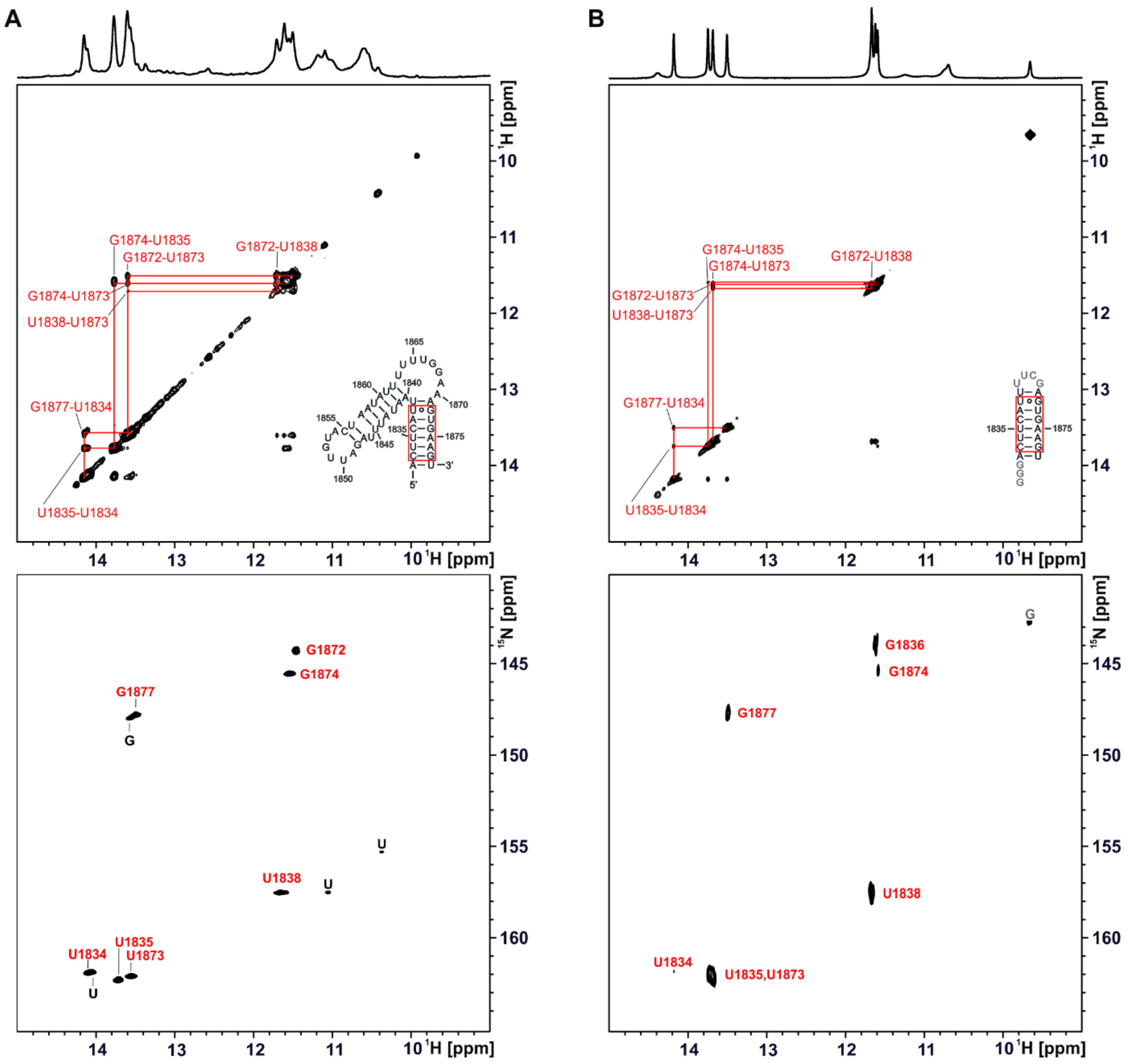

NMR analysis of the stabilized hot conformation and 23mer$^{S.lyco}$. **A)** ^{1}H,^{1}H-NOESY (Top) and 1H,^{15}N-TROSY spectrum (bottom) of the stabilized hot conformation of 75mer. The secondary structure determined experimentally is shown in the inlet with non-native G nucleotides highlighted in grey. The structural section that could be assigned is marked with a box in the secondary structure. The spectra of the RNA were recorded in 25 mM potassium phosphate buffer (pH 6.3) with 8% D_2O at 10 °C. Top: NOESY spectrum of the stabilized hot conformation of 75mer (270 µM) recorderded at 950 MHz with 3072 × 620 points and 280 scans. Bottom: TROSY spectrum of ^{15}N GU-labelled stabilized hot conformation of 75mer (30 µM) at 950 MHz with 2024x256 points and 632 scans. **B)** ^{1}H,^{1}H-NOESY (Top) and ^{1}H,^{15}N-SFHMQC spectrum (Bottom) of 23mer$^{S.lyco}$ at the 3'SS annotated with assignment. The secondary structures determined experimentally are shown in the inlet with non-native G nucleotides highlighted in grey. Structural sections that could be assigned are marked with boxes in the secondary structure. The spectra of the RNAs were recorded in 25 mM potassium phosphate buffer (pH 6.3) with 8% D_2O at 10 °C. Top: NOESY spectrum of 23mer$^{S.lyco}$ (250 µM) recorded at 950 MHz with 3072 × 768 points and 176 scans. Bottom: SFHMQC spectrum of 23mer$^{S.lyco}$ (250 µM) at 950 MHz with 2650x128 points and 2688 scans.

Supplementary Table S1:

Free energies determined with the RNAstructure RNA folding software at various temperatures. The free energies of the cold and hot conformation of the 75mer as well as the free energies of the stabilized cold conformation of the 75mer and stabilized hot conformation were determined on the RNAstructure webserver. Some energy values at certain temperatures could not be determined exactly from the constructs, as their structure was displayed slightly differently in the folding program. These deviations are given in brackets and only energies of structures that deviate by less than 4 bp are shown. With a few exceptions, all energies were obtained from folds carried out using the standard settings of the webserver. Some values could only be obtained by changing this setting (marked with *). *: to obtain the free energy, the maximum % energy difference (MFE, MEA) was set to 100%.

Folding temperature	Free energy of 75mer cold conformation	Free energy of 75mer hot conformation	Free energy of stabilized 75mer cold conformation	Free energy of stabilized hot conformation
0°C	-25.6	n.d.(*)	-30.2	-18.3
10°C	-20.3	n.d. (*)	-25.0	-15.1
15°C	-17.9	n.d. (*)	-22.6	-13.9
25°C	-13.2	n.d. (*)	-17.8	-10.8
30°C	-10.6	n.d. (*)	-15.2	-9.0
35°C	-8.5	-8.2 (structure contains 4 additional bp)	-13.2	-8.0
40°C	-5.5	-5.6	-10.2	-5.8
45°C	n.d./ -4.0 * (structure missing 2 bp)	-4.8	-8.6 (structure missing 2 bp)	-4.9
50°C	n.d./ -1.8*	-3.4	-6.5	-3.5

Supplementary Figure S11:

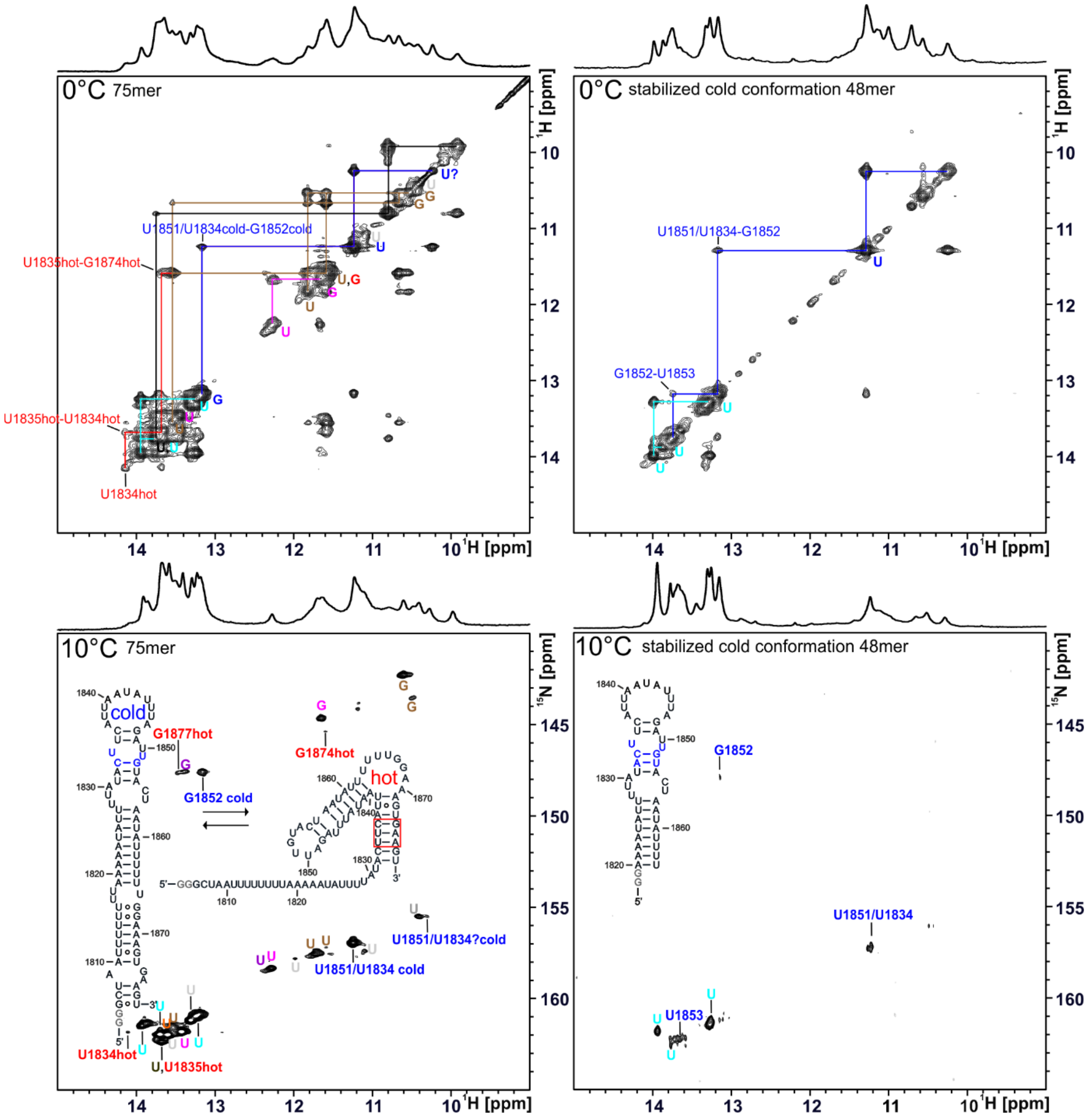

NMR analysis of 75mer and the stabilized cold conformation 48mer at 0/10°C. **Left**: ^{1}H,^{1}H-NOESY spectrum at 0°C and ^{1}H,^{15}N-TROSY spectrum at 10°C of the 75mer RNA from *S. lycopersicum* at the 3'SS. **Right**: ^{1}H,^{1}H-NOESY spectrum at 0°C and ^{1}H,^{15}N-SFHMQC at 10°C of the 48mer RNA (stabilized cold conformation of the 75mer RNA from *S. lycopersicum* at the 3'SS). The spectra of the RNAs were recorded in 25 mM potassium phosphate buffer (pH 6.3) with 8% D_2O. Left top: NOESY spectrum of the 75mer RNA (1 mM) recorded at 950 MHz with 2690 × 752 points and 120 scans. Left bottom: TROSY spectrum of 75mer RNA (200 µM) at 600 MHz with 1024x256 points and 64 scans. Right top: NOESY spectrum of the 48mer RNA (470 µM) recorded at 950 MHz with 2690 × 752 points and 120 scans. Right bottom: SFHMQC spectrum of 48mer RNA (470 µM) at 600 MHz with 2048x128 points and 2628 scans. The imino proton signals, which are presumed to originate from the hot conformation of the 75mer, are marked in red in the TROSY spectra of the 75mer. Correlations of these imino protons, which are identical to those of the stabilized hot conformation, are shown in red in the NOESY of the 75mer and are marked with a red box in the secondary structure model of the RNA. The iminoproton signals, originating from the cold conformation / stabilized cold conformation 48mer of the 75mer RNA, are marked in light and dark blue in the TROSY spectra. Some correlations of these imino protons were assigned (highlighted in blue in the secondary structure models of the RNAs).

Supplementary Figure S12:

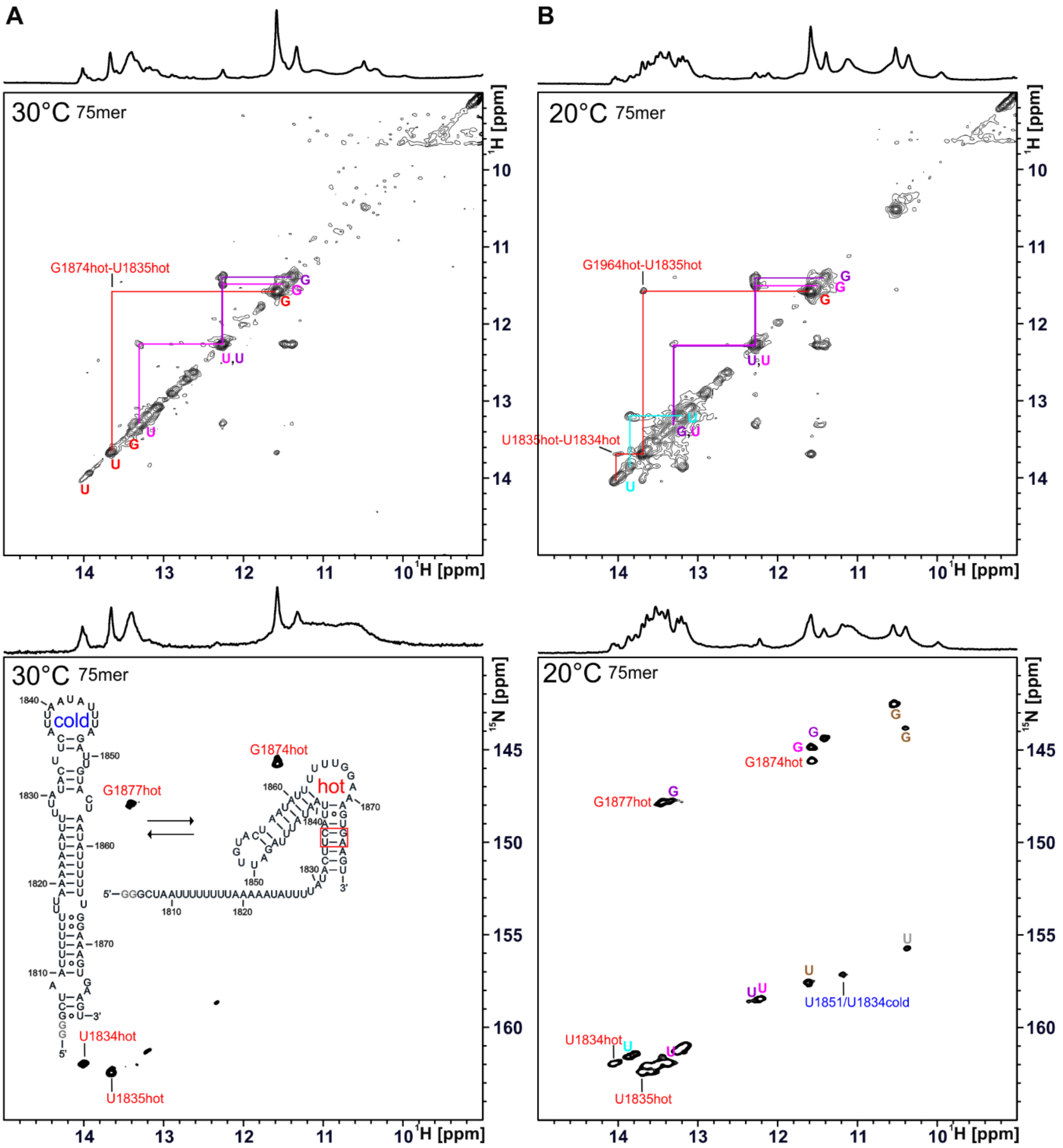

NMR analysis of 75mer at 20°C and 30°C. ^{1}H,^{1}H-NOESY spectra and ^{1}H,^{15}N-TROSY spectra of the 75mer RNA from *S. lycopersicum* at the 3'SS at 30°C (left) and 20°C (right). The spectra of the RNAs were recorded in 25 mM potassium phosphate buffer (pH 6.3) with 8% D_2O.The TROSY spectra of the RNA (200 µM) were recorded at 600 MHz with 1024x256 points and 64 scans. The NOESY spectra of the RNA (1 mM) were recorded at 950 MHz with 2690 × 752 points and 120 scans. The iminoproton signals, which are presumed to originate from the hot conformation of the 75mer RNA, are marked in red in the TROSY spectra of the 75mer. Correlations of these imino protons, which are identical to those of the stabilized hot conformation, are shown in red in the NOESY of the 75mer RNA and are marked with a red box in the secondary structure model of the RNA. The iminoproton signals, originating from the cold conformation of the 75mer, are marked in light blue and dark blue in the TROSY spectrum at 20°C of the 75mer RNA. Correlations of these imino protons, are shown in light blue in the NOESY of the 75mer RNA at 20°C.

Supplementary Figure S13:

S.galapagense
GCUAAUUUUUUUAAAAAAUAUUUUAUACUUCAUUAAUAUUUAGAUUGUACUAAUAUUUUUUGGAAAGUGAAGU
S.pimpinellifolium
GCUAAUUUUUUUAAAAAAUAUUUUAUACUUCAUUAAUAUUUAGAUUGUACUAAUAUUUUUUGGAAAGUGAAGU
S.arcanum
GCUAAUUUUUUAAAAAAAUAUUUUAUACUUCAUUAAUAUUUAGAUUGUACUAAUAUUUUUUGGAAAGUGAAGU
S.corneliomulleri
GCUAAAUUUUUUUAAAAAUAUUUUAUACUUCAUUAAUAUUUAGAUUGUACUAAUAUUUUUUGGAAAGUGAAGU
S.neorickii
GCUAAUUUUUUUAAAAAAUAUUUUAUACUUCAUUAAUAUUUAGAUUGUACUAAUAUUUUUUGGAAAGUGAAGU
S.chilense
GCUAAUUUUUUUUAAAAAUAUUUUAUACUUCAUUAAUAUUUAGAUUGUACUAAUAUUUUUUGGAAAGUGAAGU
S.chmielewskii
GCUAAUUUUUUUUAAAAAUAUUUUGUACUUCAUUAAUAUUUAGAUUGUACUAAUAUUUUUUGGAAAGUGAAGU
S.habrochaites
GCUAAAUUUUUUUAAAAUAUUUUAUACUUCAUUAAUAUUUAGAUUGUACUAAUAUUUUUUGGAAAGUGAAGU
S.peruvianum
GCUAAAUUUUUUUAAAAAUAUUUUAUACUUCAUUAGUAUUUAGAUUGUACUAAUAUUUUUUGGAAAGUGAAGU
S.lycopersicum
GCUAAUUUUUUUAAAAAAUAUUUUAUACUUCAUUAAUAUUUAGAUUGUACUAAUAUUUUUUGGAAAGUGAAGU

Sequence conservation and variation of the 75mer HsfA2 pre-mRNA fragment from *S. lycopersicum* at the 3‘SS in several tomato species. SNPs are highlighted in red and the sequence of the assigned stem of the hot conformation is highlighted in blue. According to the structure prediction, the corresponding pre-mRNA fragments of HsfA2 of the listed tomato species can undergo an identical or similar temperature-dependent conformational change, although some differ in part from SNP‘s from 75mer *S. lycopersicum*.
